# PD-L1 checkpoint inhibition and anti-CTLA-4 whole tumor cell vaccination counter adaptive immune resistance: A mouse neuroblastoma model that mimics human disease

**DOI:** 10.1371/journal.pmed.1002497

**Published:** 2018-01-29

**Authors:** Priya Srinivasan, Xiaofang Wu, Mousumi Basu, Christopher Rossi, Anthony D. Sandler

**Affiliations:** The Joseph E. Robert Jr. Center for Surgical Care and The Sheikh Zayed Institute for Pediatric Surgical Innovation, Children’s National Medical Center, George Washington University, Washington, DC, United States of America; Washington University School of Medicine, UNITED STATES

## Abstract

**Background:**

Adaptive immune resistance induces an immunosuppressive tumor environment that enables immune evasion. This phenomenon results in tumor escape with progression and metastasis. Programmed cell death-ligand 1 (PD-L1) expressed on tumors is thought to inhibit tumor-infiltrating lymphocytes (TILs) through programmed cell death 1 (PD1), enabling adaptive immune resistance. This study investigates the role of PD-L1 in both mouse and human neuroblastoma immunity. The consequence of PD-L1 inhibition is characterized in the context of an established whole tumor cell vaccine.

**Methods and findings:**

A mouse model of neuroblastoma was investigated using an Id2 knockdown whole cell vaccine in combination with checkpoint inhibition. We show that immunogenic mouse neuroblastoma acquires adaptive immune resistance by up-regulating PD-L1 expression, whereas PD-L1 is of lesser consequence in nonimmunogenic neuroblastoma tumors. Combining PD-L1 checkpoint inhibition with whole tumor cell/anti-CTLA-4 vaccination enhanced tumor cell killing, cured mice with established tumors, and induced long-term immune memory (6 months). From an evaluation of patient neuroblastoma tumors, we found that the inflammatory environment of the mouse neuroblastoma mimicked human disease in which PD-L1 expression was associated directly with TILs and lower-risk tumors. High-risk patient tumors were lacking both TILs and PD-L1 expression. Although a correlation in immunity seems to exist between the mouse model and human findings, the mouse tumor model is induced and not spontaneously occurring, and furthermore, the number of both mouse and human correlates is limited.

**Conclusions:**

This study demonstrates the role PD-L1 plays in neuroblastoma’s resistance to immunity and defines the nonredundant effect of combination checkpoint inhibition with vaccine therapy in a mouse model. High-risk, nonimmunogenic human tumors display both diminished PD-L1 expression and adaptive immune resistance. Paradoxically, high-risk tumors may be more responsive to effective vaccine therapy because of their apparent lack of adaptive immune resistance.

## Abstract 2

Anthony Sandler and colleagues reveal an approach that overcomes adaptive immune response in mouse neouroblastoma tumors, killing the tumors and providing promise for the human disease.

## Author summary

**Why was this study done?:**

- Neuroblastoma is the most common extracranial solid tumor found in children, and high-risk disease continues to have a poor prognosis despite multimodal therapy.
- A key feature of neuroblastoma tumors is their heterogeneity and the presence of an immunosuppressive tumor microenvironment that helps evade antitumor immunity.
- Checkpoint inhibitors put “the brakes” on the immune system, thus dampening the host’s immune response.
- The role of programmed cell death-ligand 1 (PD-L1), a checkpoint inhibitor, in generating immune resistance that adapts to immune conditions in neuroblastoma is not well studied.
- Early studies of checkpoint inhibition in neuroblastoma have not been successful; thus, the benefit of blocking the programmed cell death 1 (PD1)/PD-L1 axis for enhanced immunotherapy needs to be better understood.

**What did the researchers do and find?:**

- The checkpoint inhibitor PD-L1 is expressed on mouse and human neuroblastoma and is up-regulated in response to interferon gamma (IFNγ) exposure or infiltration of T cells into the tumor.
- Inhibiting PD-L1 in the context of tumor vaccination combined with another checkpoint inhibitor, anti-CTLA-4, cures established mouse neuroblastoma tumors.
- In a nonimmunogenic, aggressive mouse neuroblastoma model (AgN2a), PD-L1 expression is neither significant nor up-regulated in response to IFNγ and T-cell infiltrates, making the tumor more susceptible to vaccine therapy.
- Tumor infiltration of T cells and PD-L1 expression are associated with risk stratification in human tumors in which high-risk disease is lacking both tumor-infiltrating lymphocytes and PD-L1 expression in pretreatment tumor biopsies.

**What do these findings mean?:**

- PD-L1 is a nonredundant checkpoint in tumor vaccine therapy, and blocking this checkpoint in combination with whole cell vaccination and CTLA-4 inhibition enables enhanced antitumor T-cell killing.
- Combining appropriate checkpoint inhibitors with tumor vaccine therapy can overcome immune resistance in immunogenic neuroblastoma tumors.
- The use of checkpoint inhibitors alone in the treatment of nonimmunogenic (high-risk) neuroblastoma will most likely have little benefit.

## Introduction

Neuroblastoma is the most common extracranial solid tumor found in children and continues to have a poor prognosis in cases of high-risk disease, despite multimodal therapy [1–3]. Immunotherapy in the form of either targeted antibodies or checkpoint inhibitors is changing cancer treatment, but many tumors are either nonimmunogenic or co-opt immunosuppressive pathways that evade immune-mediated clearance.

The immune system has inhibitory pathways that maintain self-tolerance and modulate immunity to prevent autoimmune side effects [4]. These inhibitory pathways, known as “checkpoints,” are also exploited by tumors to dampen and evade antitumor immunity. CTLA-4 is a key molecule expressed on the surface of T cells. It down-regulates the T cell’s response when the immune system is activated; hence, blocking its function, either alone or in combination with other therapies, leads to improved T-cell activation and expansion [5–7]. Programmed cell death 1 (PD1) is another immune checkpoint receptor and is more broadly expressed on T cells than CTLA-4 [8, 9]. It is proposed to function downstream in the immune response, limiting the activity of T cells in peripheral tissues that express PD-L1, and thus reduce autoimmunity [10–12]. PD-L1 is expressed on the surface of many tumors as well, but the benefit of blocking the PD1/PD-L1 axis for immunotherapy is not defined in neuroblastoma.

In order to induce effective immunity against a tumor, increased immunogenicity of the tumor itself is necessary. We recently reported that Id2 knockdown of mouse neuroblastoma (Id2kd-N2a) cells are rejected by most mice following inoculation and that the same mice then fail to grow tumors when subsequently rechallenged with wild-type Neuro2a cells. Antibody depletion of CD8^+^ cells or immune-incompetent mice grow Id2kd tumors avidly, validating the concept that Id2 knockdown confers tumor cell immunogenicity in immune-competent hosts. Thus, Id2kd tumor cells can be used as whole cell vaccines, in which the altered tumor cells themselves are administered back to the host as a vaccine to induce antitumor immunity. Acting in concert with a costimulatory CTLA-4 checkpoint inhibitor, Id2kd-N2a whole tumor cell vaccination generated a potent tumor-specific T-cell response, capable of eradicating established tumors in 60% of mice [13, 14]. Surprisingly, in the same strain of mice, this vaccine approach was even more effective in a nonimmunogenic, aggressive (AgN2a) model, suggesting a less immunosuppressive tumor microenvironment [14]. When CTLA-4 was used alone without vaccination in the wild-type (WT) N2a model or the AgN2a model, only 40% and 0% of mice were cured of tumor, respectively [14].

This study investigates the role of PD-L1 checkpoint inhibition in neuroblastoma. We show that PD-L1 is expressed on mouse and human neuroblastoma and is up-regulated following interferon gamma (IFNγ) treatment or T-cell tumor infiltration. CTLA-4 blockade plus Id2kd vaccination induces tumor specific T-cell expansion and tumor infiltration in mice, in which the infiltrating CD8 T cells are characterized by PD1 expression. The combination of Id2kd-N2a cell vaccination with anti CTLA-4 plus anti PD-L1 antibody treatment proved to be highly effective, even against established neuroblastoma tumors, resulting in cure of treated mice (*n* = 16) as well as long-term immune memory (6 months). In a nonimmunogenic, aggressive neuroblastoma model (AgN2a), PD-L1 expression is neither significant nor up-regulated in response to IFNγ and T-cell infiltrates, making the tumor more susceptible to vaccine therapy. Characteristically, tumor infiltration of T cells and PD-L1 expression seem to also be associated with risk stratification in human neuroblastoma tumors. Low- and intermediate-risk tumors have abundant infiltrating T cells that are surrounded with high PD-L1 tumor expression, while high-risk tumors lack significant T-cell infiltrates and PD-L1 expression.

## Materials and methods

### Animals

Female A/J mice aged 6 weeks were purchased from Jackson Laboratories (Bar Harbor, Maine, United States). The animals were acclimated for 4–5 days prior to tumor challenge. All procedures were approved by the Institutional Animal Care and Use Committee (IACUC) of Children’s National Medical Center, Washington, DC.

### Cells

The murine neuroblastoma cell line Neuro2a (N2a) is derived from an A/J mouse and was purchased from the American Type Culture Collection (ATCC, Manassas, Virginia, US). The aggressive N2a subclone AgN2a was derived from repeated in vivo passaging of these cells as described previously [14]. The cells were maintained in Dulbecco’s Modified Essential Medium (DMEM) supplemented with 1% penicillin-streptomycin (Invitrogen, Carlsbad, California, US) and 10% fetal bovine serum (Gemini Bioproducts, Sacramento, California, US). Mouse splenocytes were cultured in RPMI medium supplemented with 2 mM L-glutamine, 10% fetal bovine serum, and 1% penicillin-streptomycin. Cells were grown at 37°C under 5% CO_2_.

Human neuroblastoma cell lines IMR-32, SK-N-SH, and SH-SY5Y were obtained from the ATCC. IMR-32 and SK-N-SH cells were grown in ATTC Eagle’s Minimum Essential Medium (EMEM) supplemented with 10% FBS. SH-SY5Y cells were cultured in ATCC EMEM mixed 1:1 with F12 medium, and FBS was added to a final concentration of 10%.

### Whole tumor cell vaccine

Id2kd-N2a whole tumor cells were generated as described previously [14]. The anchorage-dependent Neuro2a cells were transduced with Id2-shRNA expressing lentiviral particles containing a puromycin resistance gene (Santa Cruz Biotechnology, Santa Cruz, California, US) for stable knockdown of Id2. The stable clones expressing the Id2-shRNA (Id2kd-N2a) were selected using puromycin according to the manufacturer’s instructions.

### Specimens and patient demographics

Human specimens were obtained from 13 patients diagnosed with low-risk (*n* = 3), intermediate-risk (*n* = 5), and high-risk (*n* = 5) neuroblastoma. Diagnosis and staging were performed according to Children’s Oncology Group (COG) protocols. Biopsies were taken at the time of diagnosis and prior to initiation of any therapy. Specimen collection was obtained after appropriate research consents (and assents when applicable) and was approved by the Institutional Review Board, CNMC, Washington, DC (Pro00004284).

### Antibodies and reagents

Anti (α)-mouse CTLA-4, α-mouse PD-L1, and mouse IgG2b isotype antibodies were purchased from BioXCell (West Lebanon, New Hampshire, US). Mouse α-CD4 APC, α-CD8 PerCP Cy5.5, α-PD-L1, and purified α-mouse CD3 were bought from BD Biosciences (San Jose, California, US). Mouse α-CD45 PE, α-PD1 FITC, α-TIM3 APC, and α-LAG3 APC were purchased from eBioscience and Biolegend (San Diego, California). α-mouse and α-human recombinant IFNγ was purchased from Peprotech (Rocky Hill, New Jersey, US).

### Mouse neuroblastoma therapy models

A/J mice were injected subcutaneously (s.c.) in the right flank with 1 × 10^6^ freshly prepared tumor (Neuro2a or AgN2a or N2a-luc) cells in 100 μl phosphate-buffered saline (PBS) on day 0. One million Id2kd-N2a cells were injected (s.c.) into the left flank of each mouse on day 5 and again on day 12 as a whole cell vaccine. The mice usually developed tumors of 5 mm in size on the right flank by day 6. Anti-CTLA-4 and anti-PD-L1, each at a dose of 100 μg/mouse/time point, were administered intraperitoneally on days 5, 8, and 11. Mice were monitored daily following tumor inoculation. Tumor growth was recorded on alternate days by measuring the diameter in 2 dimensions using a caliper and by imaging the mice for tumor bioluminescence using IVIS Lumina III (Perkin Elmer, Houston, Texas, US) when appropriate. Tumor volume was calculated using the following formula: (large diameter × small diameter)^2^ × 0.52. A tumor size of 20 mm in diameter in any dimension was designated as the endpoint, and mice were euthanized at that time. Euthanasia was achieved through cervical dislocation after CO_2_ narcosis. If a tumor impaired the mobility of an animal, became ulcerated, or appeared infected, or a mouse displayed signs of “sick mouse posture,” the mouse was euthanized. Food was provided on the cage floor when the tumor size reached 15 mm in diameter. All the procedures are approved by the IACUC at CNMC and are in accordance with the humane care of research animals.

### Isolation of tumor-infiltrating lymphocytes and splenocytes

Mouse tumors were harvested, mechanically disrupted, and then digested with a cocktail of collagenase I, dispase II, and DNase 1 (Sigma Aldrich, Missouri, US) as per the method described previously [14]. CD8^+^ T cells were isolated from the tumor digest by positive selection using the mouse CD8a^+^ T-cell isolation kit (Miltenyi Biotec, San Diego, California, US).

Spleens were collected from mice euthanized by CO_2_ narcosis and cervical dislocation. Spleens were pulverized through a 40-μm mesh cell strainer and treated with ACK lysing buffer to remove erythrocytes before being cultured in RPMI medium.

### Characterization of mouse tumors by immunofluorescence (IF)

Mouse tumors were excised either when they reached 10 mm or when they started to shrink following vaccine therapy. Specimens were fixed in 10% neutral buffered formalin (pH 6.8–7.2; Richard-Allan Scientific, Kalamazoo, Michigan, US) for paraffin embedding and sectioning. Five μm tissue sections were cut with a microtome, and sample processing and IF staining were performed as previously described using the following primary antibodies: CD3 rabbit anti-mouse mAb (1:100, ab16669, Abcam, Cambridge, Massachusetts, US) and PD-L1 goat anti-mouse polyclonal Ab (1:20, AF1019, R&D Systems, Minneapolis, Minnesota, US). Isotype-matched antibodies were used for negative controls. Sections were mounted with ProLong Diamond Antifade Mountant with DAPI (Thermo Fisher Scientific, Halethorpe, Maryland, US).

### Flow cytometry

Cells from mouse tumor digests and mouse splenocytes were stained with the fluorescently conjugated antibodies described above. Flow cytometry was done using a Becton Dickinson/Cytek FACSCalibur (BD Biosciences, San Jose, California, US). Data were analyzed using the FlowJo program (Treestar, Ashland, Oregon, US).

### IFNγ measurement

A total of 2 × 10^4^ freshly isolated mouse splenocytes were plated in a volume of 200 μl per well of 96-well round bottom plates. Splenocytes were stimulated with 2 × 10^5^ WT N2a cells and 1.0 μg/ml α-CD3. WT N2a was blocked with 10 μg/ml α-mouse PD-L1, α-mouse CTLA-4 antibody, or IgG2b isotype control for 24 hours prior to interaction with splenocytes. Blocking was continued at the same concentration during the interaction with the splenocytes. Plates were incubated at 37°C under 5% CO2 for 48 hours. Supernatants were collected from triplicate wells, and IFNγ was assayed using the Ready-set-go mouse IFNγ ELISA kit from Ebioscience (San Diego, California, US). Readings were measured at 450 nm using the EnSpire 2300 Multilabel plate reader (Perkin Elmer, Waltham, Massachusetts, US).

An Enzyme-Linked ImmunoSpot (ELISpot) assay was performed in duplicate under the same cell conditions listed above, using the mouse IFNγ ELISpot Basic kit (Mabtech, Cincinnati, Ohio, US). Counting of spots and data analysis were carried out by ZellNet Consulting (Fort Lee, New Jersey, US).

### Cytotoxicity assay

A modified flow-cytometry-based cytotoxicity assay detecting the presence of activated caspase 3 in target cells was performed. WT N2a target cells were labeled with CellTrace Far Red stain (Invitrogen, Carlsbad, California, US). Tumor-infiltrating CD8^+^ T cells (CD8 TILs) were isolated from established tumors of A/J mice following complete vaccination with Id2Kd N2a, plus anti-CTLA-4+anti-PD-L1. Effector cells to target cells were incubated at a 1:20 ratio for 3 hours at 37°C. The cells were then fixed and stained with PE-conjugated activated caspase 3 antibody (BD Biosciences, San Diego, California, US). Target cells with no added effectors were used to determine spontaneous death. Drug-induced killing of tumor targets was determined by incubation with 1 μm campothecin and 1 μm staurosporine for 3 hours at 37°C. Gating and flow cytometry analysis was carried out according to the protocol described [15].

### Fluorescent multiplex immunohistochemistry

Five-micron-thick formalin-fixed paraffin-embedded (FFPE) human neuroblastoma tissue sections were deparaffinized in xylene and hydrated with graded alcohol and distilled water. Antigen retrieval was performed in EDTA unmasking solution (cell signaling) using a vegetable steamer for 15 minutes. This was followed by blocking of endogenous peroxidase activity with 3% hydrogen peroxide for 10 minutes (Sigma, Bellefonte, Pennsylvania, US). After rinsing the slides in PBS, the slides were incubated with CD3ε (D7A6E) XP Rabbit anti-human mAb (1:250, #85061, Cell signaling) for 1 hour at room temperature (RT). The antigen-antibody reaction was boosted by SignalStain Boost Detection Reagent for 30 minutes. Following a wash, the slides were incubated with the Tyramide (TSA)-plus Cyanine 3 (NEL744001KT, PerkinElmer, Life Technologies) at 1:100 dilution for 10 minutes. For double staining with PD-L1, the slides were brought to a boil, the antibody-antigen reaction for CD3 was stripped in 10 mM sodium citrate buffer (PH = 6, #14746, cell signaling) for 10 minutes, and then repeat staining/boosting/detection was performed using PD-L1 (E1L3N) XP Rabbit anti-human mAb (1:250, #77563, Cell signaling) and TSA-plus FITC (NEL741001KT, PerkinElmer, Life Technologies). Sections were then mounted with ProLong Diamond Antifade Mountant with DAPI (Thermo Fisher Scientific).

### Confocal microscopy imaging

IF-stained markers were observed with individual sections (*xy* plane). Confocal images were acquired with a Zeiss LSM 510 confocal microscope (Carl Zeiss MicroImaging, Thornwood, New York, US) using Zen 2010 Light Edition acquisition software. Images were taken at magnifications of 100× and 630× under oil immersion.

### Quantitative analysis of PD-L1 and CD3 expression in human tissue

Ten to fifteen randomly selected fields in each stained specimen were imaged under 100× magnification. Quantification of fluorescent intensity was achieved using Olympus cellSens imaging software (version 1.7). The fluorescent intensity in each field was measured using a manual threshold setting. Measurements were made by the same person, and this individual was blind to the identity of the specimen. Data were presented as the mean fluorescent intensity of all the fields for each specimen.

### Statistical analysis

The specific tests used to analyze each set of experiments are indicated in the figure legends. For each statistical analysis, appropriate tests were selected on the basis of whether the data was normally distributed by using the D'Agostino-Pearson normality test. Data were analyzed using an unpaired 2-tailed Student *t* test for comparisons between 2 groups and 2-way repeated-measures ANOVA to compare differences between average tumor growth curves. Survival curves were calculated according to the Kaplan-Meier method; survival analyses were performed using the log-rank test. Statistical calculations were performed using GraphPad Prism software (GraphPad Software, San Diego, California, US), and the probability level of *p* < 0.05 was considered significant.

## Results

### PD-L1 is expressed on both mouse and human neuroblastoma and is up-regulated by IFNγ exposure or tumor-infiltrating T cells

**Fig 1:**
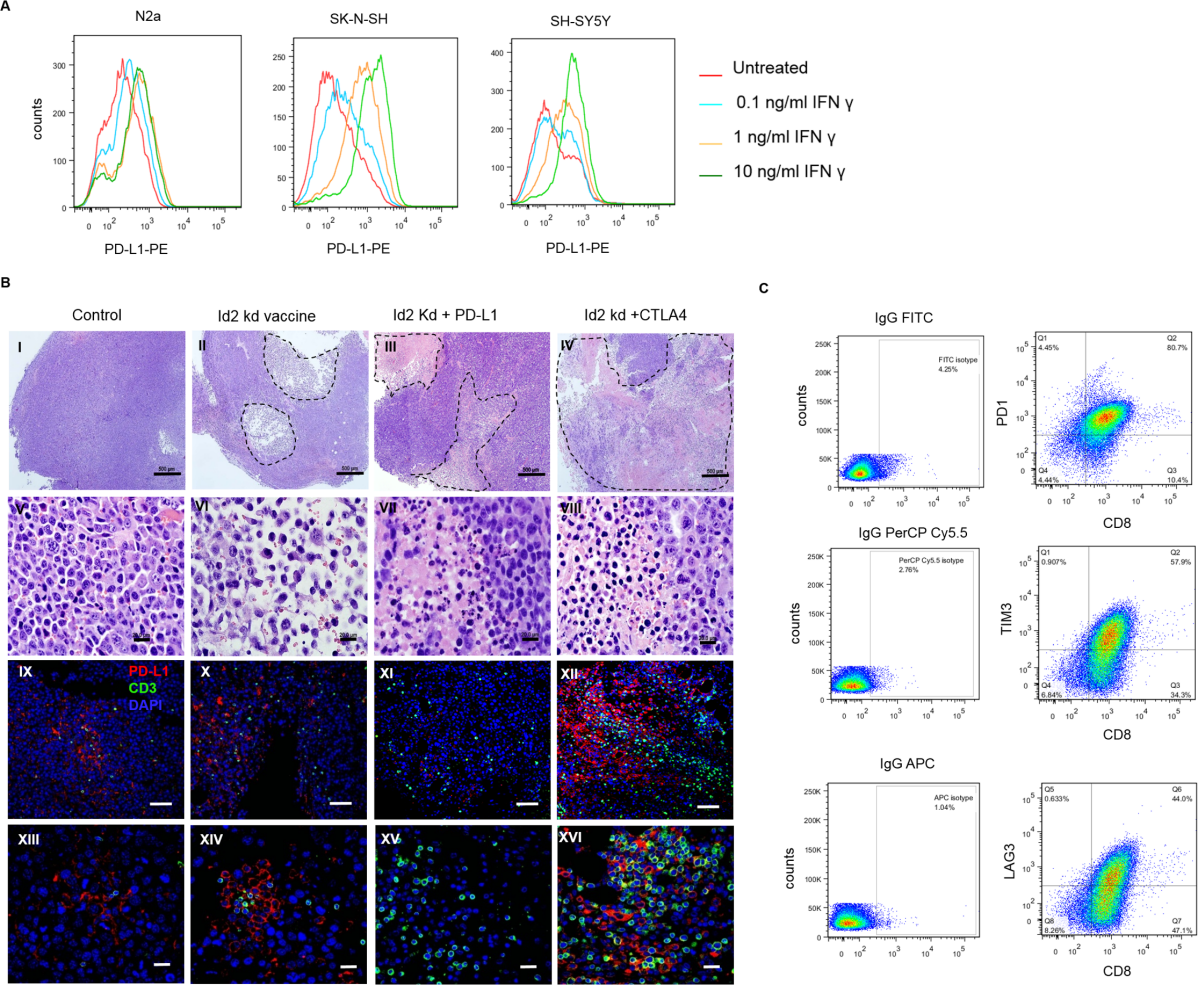
Programmed cell death-ligand 1 (PD-L1) is expressed on both mouse and human neuroblastoma cell lines and is up-regulated following 24-hour interferon gamma (IFNγ) exposure. (A) PD-L1 expression on the surface of mouse Neuro2a was analyzed by flow cytometry and is up-regulated in a dose-dependent manner (mean fluorescent intensity [MFI]). The expression of PD-L1 on human neuroblastoma cell lines SY5Y and SK-N-SH (non-NMYC amplified) had similar changes with exposure to IFNγ. (B) CD3 and PD-L1 expression in mouse neuroblastoma tumors following receipt of Id2kd vaccine with or without checkpoint blockade therapy. Representative tumors were dissected from naïve mice (I, V, IX, and XIII) and from mice after receipt of Id2kd vaccine (II, VI, X, and XIV), Id2kd plus PD-L1 antibody (III, VII, XI, and XV), and Id2kd plus CTLA-4 antibody (IV, VIII, XII, and XVI). Hematoxylin and eosin (H&E) staining (I–VIII) and immunofluorescence double staining (IX–XVI) for CD3 (green) and PD-L1 (red) were performed. The nuclei were stained with DAPI (blue). Representative micrographs from each cohort are shown. Areas of necrotic tissues are marked with black broken lines and coincide with areas of inflammatory cell infiltrates. Panel 1 (I–IV) and panel 3 (IX–XII) are 40× and 100× original magnification, respectively. The scale bar for (I–IV) is 500 μm and for (IX–XII) is 100 μm. The enlarged images in panel 2 (V–VIII) and panel 4 (XIII–XVI) are of 600× original magnification. The scale bar is 20 μm. (C) Expression of activation markers on the surface of CD8+ tumor-infiltrating lymphocytes isolated from shrinking tumors of mice treated with α-CTLA-4 plus Id2kd vaccine. Programmed cell death 1 (PD1), TIM3, and LAG3 were expressed on tumor-infiltrating lymphocytes (TILs) by flow cytometry compared with isotype controls. APC, antigen-presenting cell; FITC, fluorescein isothiocyanate; IgG, immunoglobulin G; PerCP, peridinin-chlorophyll-protein complex.

PD-L1 is detected on the mouse Neuro2a cell line, and its surface expression levels increase in a dose-dependent manner after 24 hours of stimulation with IFNγ (Fig 1A). Similarly, the expression of PD-L1 rises markedly in response to increasing doses of IFNγ in the SK-NSH and SH-SY5Y human cell lines (non-MYCN amplified cell lines) (Fig 1A). Thus, IFNγ produced by TILs may induce adaptive resistance in mouse tumors via up-regulation of PD-L1 expression.

Mouse neuroblastoma morphology and CD3^+^ TILs were examined following whole cell vaccination combined with CTLA-4 or PD-L1 blocking antibody. Fig 1B, I–IV, shows that tumors from mice vaccinated with or without checkpoint inhibitors were infiltrated with leukocytes and displayed significant tumor necrosis. Tumor necrosis was most prevalent in the group that received Id2kd vaccine plus anti-CTLA-4 antibody, which also displayed the highest level of T-cell infiltrates compared to mice from the other cohorts (Fig 1B, I–XVI). These findings indicate that the combination of immune priming (Id2kd-N2a vaccine) with immune modulation (anti-CTLA-4 antibody) potently boosts T-cell immunity. It is well known that PD-L1 expression on tissues can evade immunity by binding PD1 on T cells [11, 16]. To this end, we examined whether increased T-cell infiltration induced PD-L1 tumor cell expression. We found a dramatic increase in PD-L1 expression around tumor-infiltrating lymphocytes in the mouse tumors following Id2kd plus anti-CTLA-4 treatment. Furthermore, expression levels of PD-L1 in each experimental cohort correlated with CD3^+^ T-cell influx (Fig 1B) and seemed to be associated primarily with necrotic areas of the tumor.

CD8^+^ tumor-infiltrating T cells (TILs) were isolated from the tumors of mice treated with α-CTLA-4 plus vaccine. Flow cytometry revealed strong surface expression of PD1, TIM3, and LAG3 (Fig 1C), which are thought to suppress cell mediated antitumor immunity. The expression of these markers may indicate the exhausted phenotype of anergic T cells, but these molecules are also reported to be activated in effector T cells [17]. These findings provide the rationale that blockade of both CTLA-4 and PD-L1 might lead to improved immunotherapy by virtue of their differential targets on T-cell expansion and adaptive tumor cell resistance, respectively.

### Regression, cure, and long-term immune memory of established neuroblastoma tumors with combination therapy

**Fig 2:**
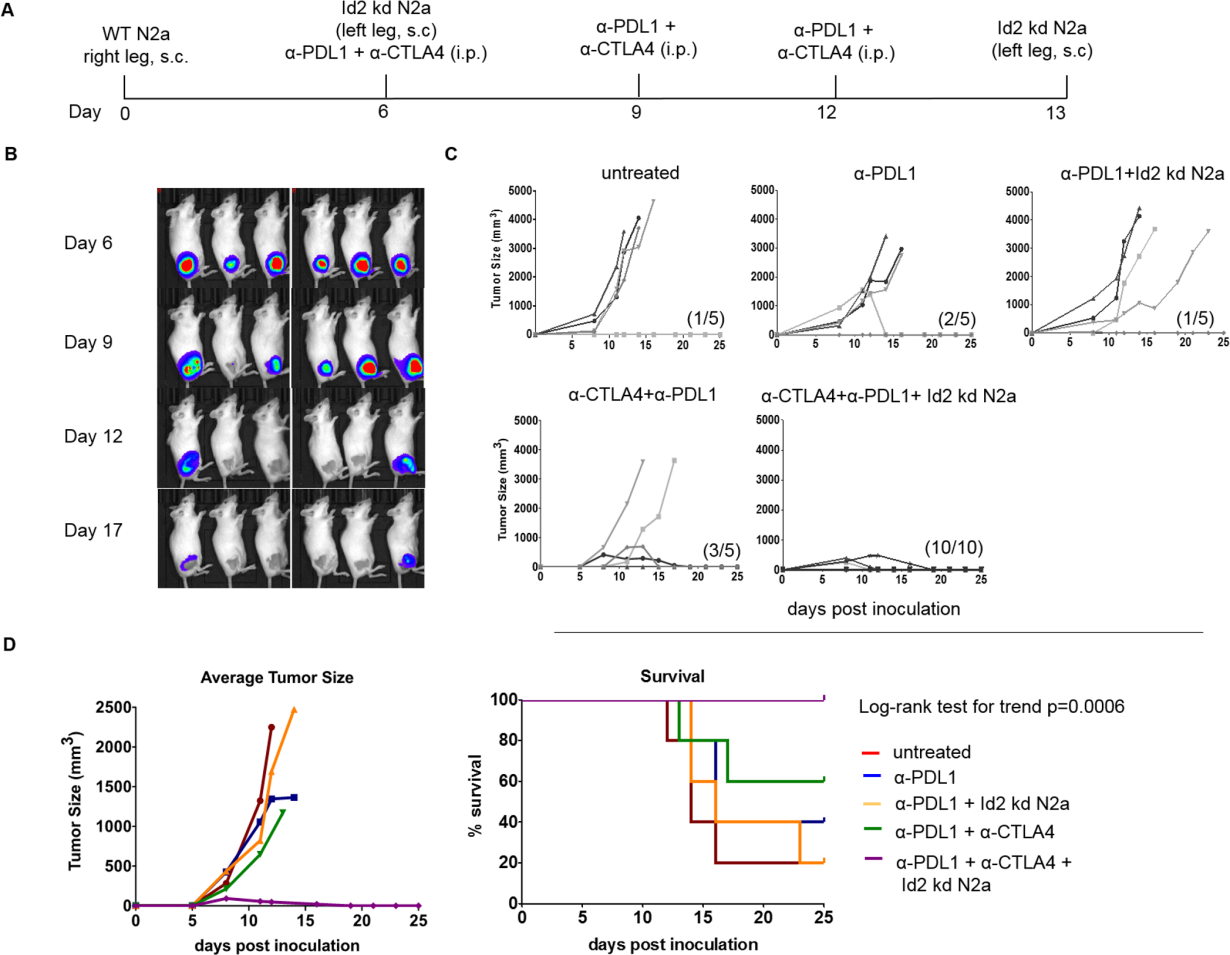
Combined vaccination with immunotherapy cures large established tumors and provides protective immunity. A/J mice were inoculated with 1 × 10^6^ wild-type (WT) N2a, and once tumors were established, the mice were then vaccinated with various combinations of Id2kd-N2a, α-CTLA-4, and α-PD-L1 blocking antibodies. (A) This panel depicts the vaccination protocol and timeline. (B) Tumor eradication in vaccinated mice (*n* = 6) as detected by chemiluminescent imaging. (C) Tumor growth in various treatment groups following vaccination. Ten out of 10 mice were cured of tumors when Id2kd-N2a vaccine was combined with inhibition of CTLA-4 and programmed cell death-ligand 1 (PD-L1) checkpoints. The graphs depict individual tumor growth over time and cure in parenthesis. (D) Average tumor growth (left panel) and survival (right) are markedly improved in the group receiving a combination of Id2kd N2a, α-CTLA-4, and α-PD-L1 when compared with other treatments (*p* = 0.0007 for average tumor growth in untreated versus α-PD-L1+α-CTLA-4+Id2kd N2a, *p* = 0.0005 for WT N2a +α-PD-L1 versus full combination, *p* = 0.0025 for WT N2a +α-PD-L1+Id2kd N2a versus full combination, 2-way repeated measures ANOVA analysis, left panel; right panel *p* = 0.0006 for survival trend, log-rank test; *p* = 0.0007 in untreated control versus α-PD-L1+α-CTLA-4+Id2kd N2a, *p* = 0.007 in α-PD-L1 versus combination, *p* = 0.0008 in α-PD-L1+Id2kd N2a versus combination, *p* = 0.0034 in α-PD-L1+α-CTLA-4 versus combination treatment, log-rank test).

We tested our whole cell vaccine strategy in the context of both CTLA-4 and PD-L1 inhibition in a model using chemiluminescent Neuro2a cells (Fig 2A and 2B). Tumors were completely eradicated in all 6 mice that received the complete vaccination. In order to rule out the possibility of additional antigenicity induced by introducing chemiluminescence into the Neuro2a cells, the study was repeated using the regular Neuro2a cell line. Vaccine, anti-CTLA-4, or anti-PD-L1 alone or in combination independently with vaccination had modest effects on established tumor growth, while the combination of both anti-CTLA-4 and anti-PD-L1 without vaccination cured 60% of mice (Fig 2C). When the vaccine was combined with both CTLA-4 and PD-L1 inhibition, all mice (*n* = 16 in both studies) were cured of their tumors (Fig 2B, 2C and 2D) and remained tumor free for 6 months in follow-up (log-rank test for survival, *p* = 0.0006) (Fig 2D, right panel). Average tumor growth curves also showed significant differences for treatment when the combination of vaccine with anti-PD-L1 and anti-CTLA-4 was compared to control (Fig 2D, left panel; *p* = 0.0007, 2-way repeated measures ANOVA). These observations demonstrate the benefit of combination checkpoint therapy in which CTLA-4 inhibition expands tumor-infiltrating lymphocytes, while PD-L1 inhibition counters adaptive resistance at the tumor site.

**Fig 3:**
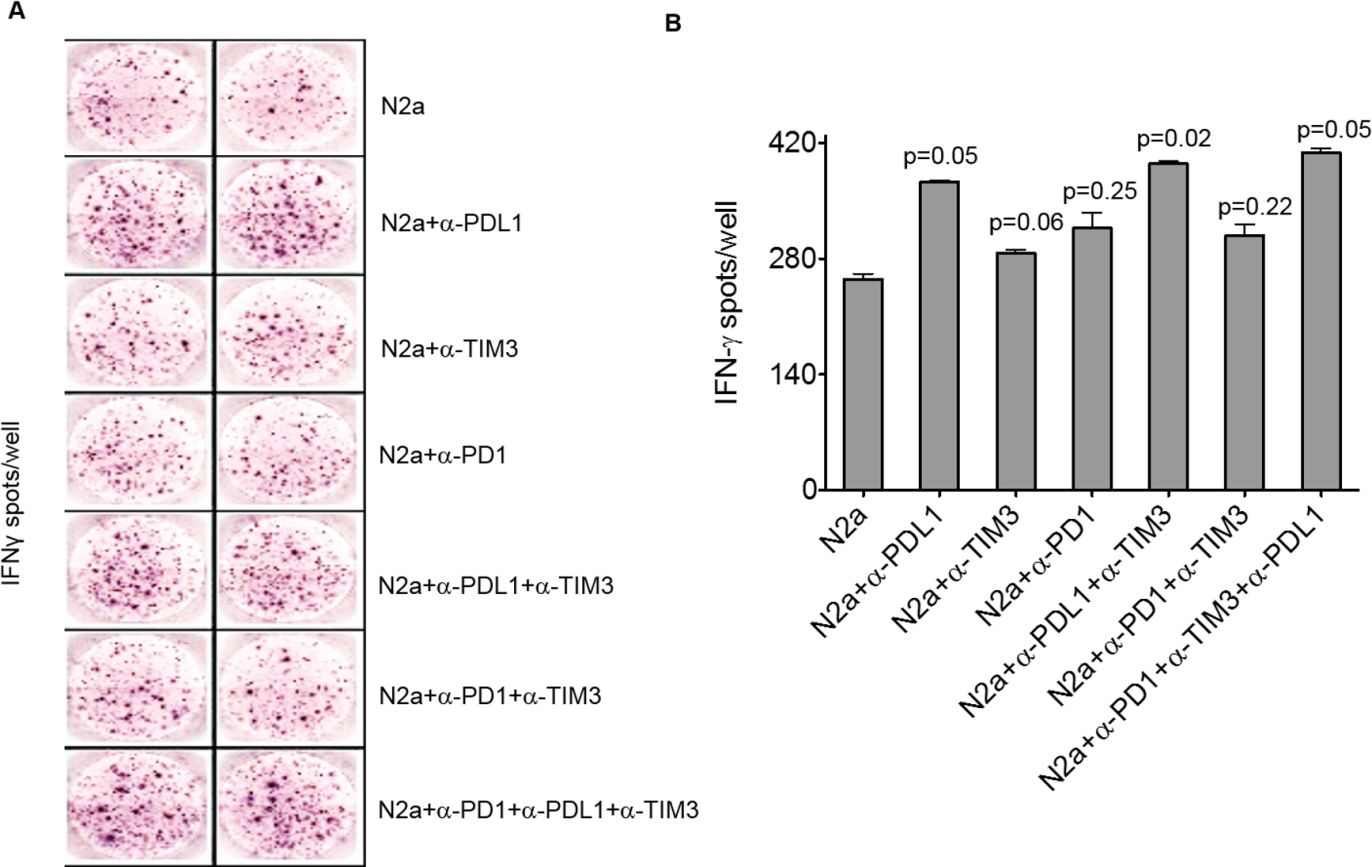
Programmed cell death-ligand 1 (PD-L1) blockade boosts interferon gamma (IFNγ) production of tumor-infiltrating lymphocytes (TILs) in vitro. CD8+ TILs isolated from tumors of mice treated with α-CTLA-4+vaccine were cocultured with wild-type (WT) N2a cells at a 10:1 ratio, for 40 hours in an IFNγ Enzyme-Linked ImmunoSpot (ELISpot) assay. Where indicated, N2a cells were blocked with 10 μg/ml α-PD-L1 for 24 hours prior to coculture, and blocking was maintained during the assay. Also as indicated, α-PD1 and α-TIM3 were added to the reactions at 10 μg/ml for the duration of the assay. Panel A shows actual IFNγ spots/well imaged from ELISpot assay in duplicate. Panel B graphs enumerated spots captured from an ELISpot reader in which each spot corresponds to a T cell producing IFNγ (unpaired 2-tailed Student *t* test, *p* < 0.0485 where significant, *p* > 0.0595 where not significant).

Neuro2a cells were treated with α-PD-L1 antibody to block surface expression and incubated with TILs isolated from the tumors of mice treated with α-CTLA-4 plus vaccine. Checkpoint blockers α-PD1 and α-TIM3 were also added as indicated, as these checkpoints were detected on TILs by flow cytometry. ELISpot analysis was performed, and a significant increase in IFNγ spots per well was observed only when α-PD-L1 was blocked, when compared to controls (Fig 3A; anti-PD-L1 alone [*p* = 0.05], anti-PD-L1 plus anti-TIM3 [*p* = 0.02]; combined blockade with anti-PD1, PD-L1 and TIM3 [*p* = 0.05]). In the absence of PD-L1 blocking, α-PD1 and/or anti-TIM3 did not enhance IFNγ production (unpaired 2-tailed Student *t* test) (Fig 3B).

**Fig 4:**
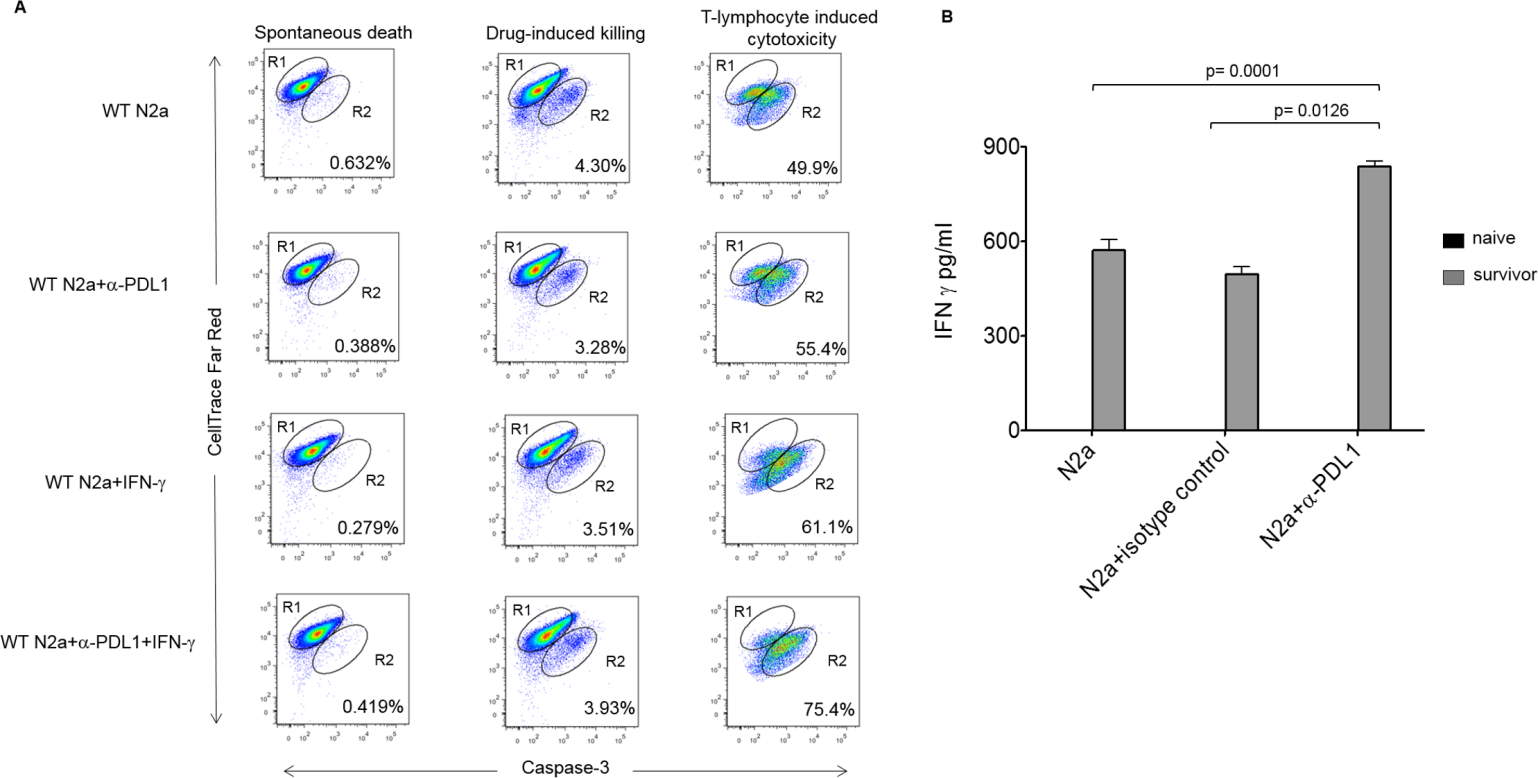
Enhanced effector function of tumor-infiltrating lymphocytes (TILs) and memory response in survivors with inhibition of programmed cell death-ligand 1 (PD-L1). (A) Modified T-cell cytotoxicity assay by caspase-3 cleavage assay. Interferon gamma (IFNγ) was used to up-regulate PD-L1 in wild-type (WT) cells. R1 represents the labeled tumor target cells, while R2 is the percentage of target cells positive for activated caspase-3. Drug cytotoxicity was induced by a combination of 1 μm staurosporine and 1 μm camptothecin, incubated for the same time as the other reactions. (B) Long-term memory response of survivors. Splenocytes were isolated from mice at 6 months following cure with α-PD-L1+α-CTLA-4 plus vaccine, as well as from naïve mice. N2a cells were cocultured with splenocytes at a 1:10 ratio for 48 hours. Supernatants were tested for IFNγ expression levels by ELISA. Naïve controls produced no detectable IFNγ (Student *t* test, *p* = 0.01; splenocyte IFNγ level with α-PD-L1 blockade).

Subsequently, TILs were collected from mouse tumors at completion of the full vaccine protocol. TILs were cultured with WT Neuro2a, and a modified flow-cytometry-based cytotoxicity assay detecting the presence of activated caspase 3 in target cells was performed. Effector:target ratios of 20:1 were used for 3 hours of coculture. In WT controls, 49.3% of tumor cells underwent apoptosis, while in PD-L1 blocked targets, 73.9% of targeted tumor cells underwent apoptosis (Fig 4A). These results confirm that effector T-cell function against neuroblastoma tumor cells is enhanced with PD-L1 blockade.

Splenocytes isolated from naïve mice and from mice that were 6-month survivors following complete vaccination with anti-PD-L1, anti-CTLA-4, and whole cell Id2kd vaccine were cocultured with Neuro2a cells in vitro. IFNγ production detected by ELISA showed potent immune memory (*p* = 0.0126 for WT N2a cocultured with splenocytes from survivors compared with N2a blocked with α-PD-L1, and *p* = 0.0001 when isotype control was compared to α-PD-L1, 2-tailed unpaired Student *t* test), whereas IFNγ responses were not detected at all in naïve splenocytes (Fig 4B). Furthermore, survivors of vaccinated mice rechallenged with WT tumor cells rejected the challenge and failed to grow tumors. Therapeutic vaccination not only cleared established tumors but also induced long-term immune memory.

### PD-L1 expression is reduced in nonimmunogenic, aggressive cell lines and tumors

**Fig 5:**
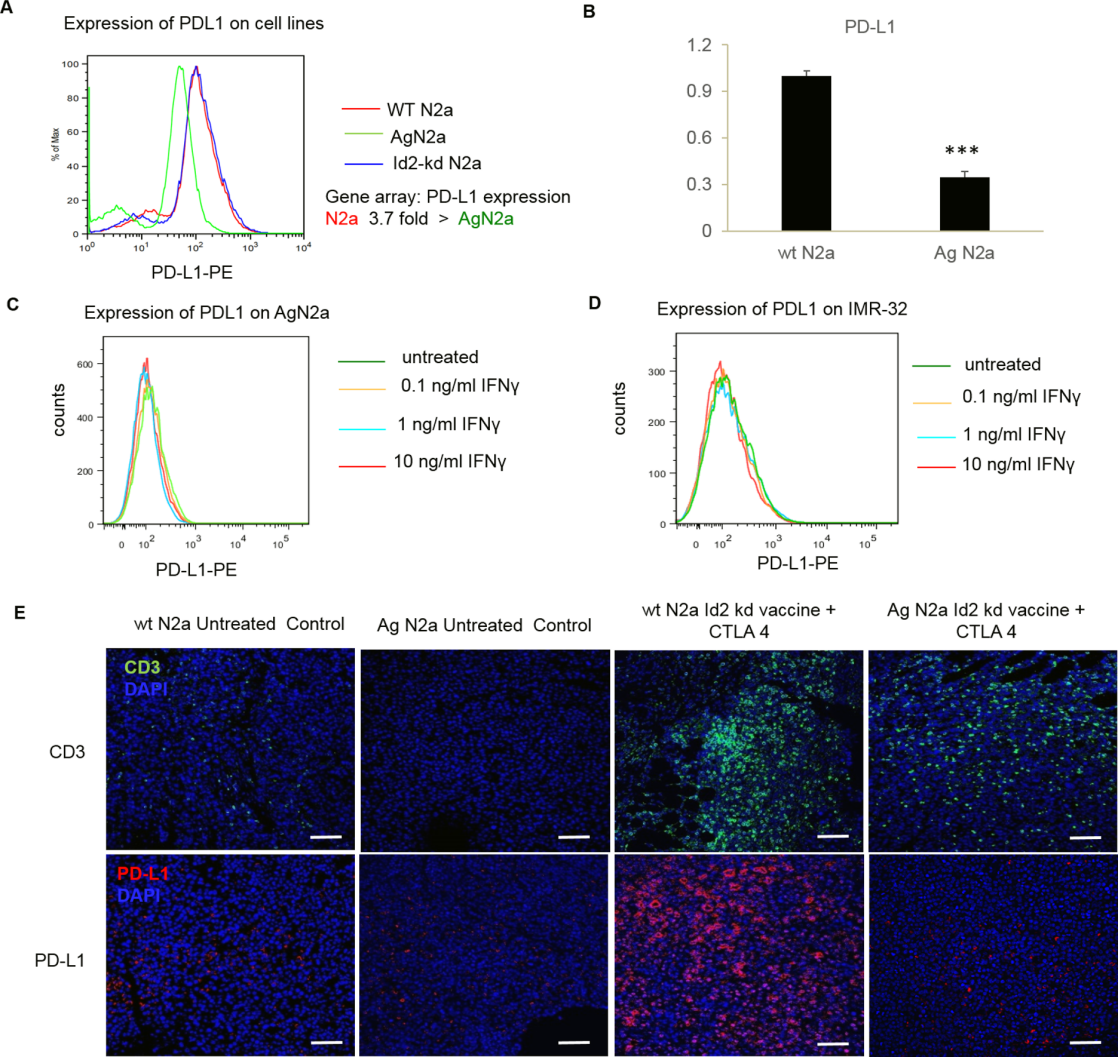
**Programmed cell death-ligand 1 (PD-L1) is expressed at 3.7-fold-lower levels on aggressive mouse neuroblastoma cell line AgN2a, as evidenced by flow cytometry (A) and real-time quantitative PCR (RT-qPCR) (*p* < 0.005, Student *t* test) (B).** (C) Exposure to even high levels of interferon gamma (IFNγ) does not up-regulate PD-L1 on AgN2a at 24 hours. (D) Similarly, the human NMYC-amplified IMR-32 cell line failed to up-regulate PD-L1 expression, unlike the other non-NMYC-amplified cell lines tested. (Fig 1). (E) Both CD3 (green) and PD-L1 (red) expression were examined by immunofluorescence (IF) staining and confocal microscopy in WT 2a and AgN2a mouse tumors at baseline and following vaccination. Representative tumors were obtained from naïve mice and mice following receipt of Id2kd vaccine plus CTLA-4 antibody alone. The nuclei were stained with DAPI (blue). Tissue sections were imaged at 200× original magnification, and the scale bar is 50 μm. IF staining demonstrates the minimal PD-L1 expression AgN2a tumors compared to WT N2a tumors, which may explain the sensitivity of AgN2a to vaccine and anti-CTLA-4 alone, without anti-PD-L1 therapy.

We previously reported that tumor vaccination plus anti-CTLA-4 antibody was surprisingly more effective in an aggressive mouse cell line (AgN2a) (90% cure) than in the wild-type Neuro2a cell line (60% cure) [14]. A possible explanation may be the differential constitutive expression of PD-L1 by tumor cells. We thus examined PD-L1 expression in both mouse and human cell lines, comparing mouse WT (Neuro2a) to aggressive (AgN2a) neuroblastoma and human non-MYCN-amplified SK-N-SH to MYCN-amplified IMR-32 cell lines [18]. MYCN amplification correlates with clinically high-risk, aggressive disease. The response of PD-L1 expression to IFNγ stimulation was also determined. Gene array data revealed a 3.5-fold-higher level of constitutive PD-L1 expression in WT N2a than in AgN2a, and this difference was verified with quantitative real-time PCR (RT-PCR) and flow cytometry (Fig 5A, 5B and 5C). The aggressive mouse AgN2a and the MYCN-amplified IMR-32 human cell lines failed to up-regulate PD-L1 following IFNγ treatment even at the highest concentrations tested (Fig 5C and 5D and Fig 1A).

To determine whether these observations of diminished PD-L1 expression in aggressive nonimmunogenic cell lines held true for growing tumors in vivo, we examined both the T-cell infiltrates and PD-L1 expression in AgN2a mouse tumors at baseline and following vaccination. Fluorescent microscopy showed no T-cell infiltration with minimal PD-L1 expression in untreated tumors, and despite a moderate influx of CD3^+^ T cells following complete vaccination, the extent of induced PD-L1 expression was markedly reduced when compared to WT Neuro2a tumors sampled after vaccination (Fig 5E).

Nonimmunogenic neuroblastoma does not up-regulate PD-L1 inhibitory pathways like immunogenic mouse neuroblastoma does. Paradoxically, this diminished adaptive resistance in aggressive nonimmunogenic tumors may enable more effective antitumor immunity.

### Immunogenic human tumors also acquire PD-L1 adaptive resistance, which is associated with risk stratification

**Fig 6:**
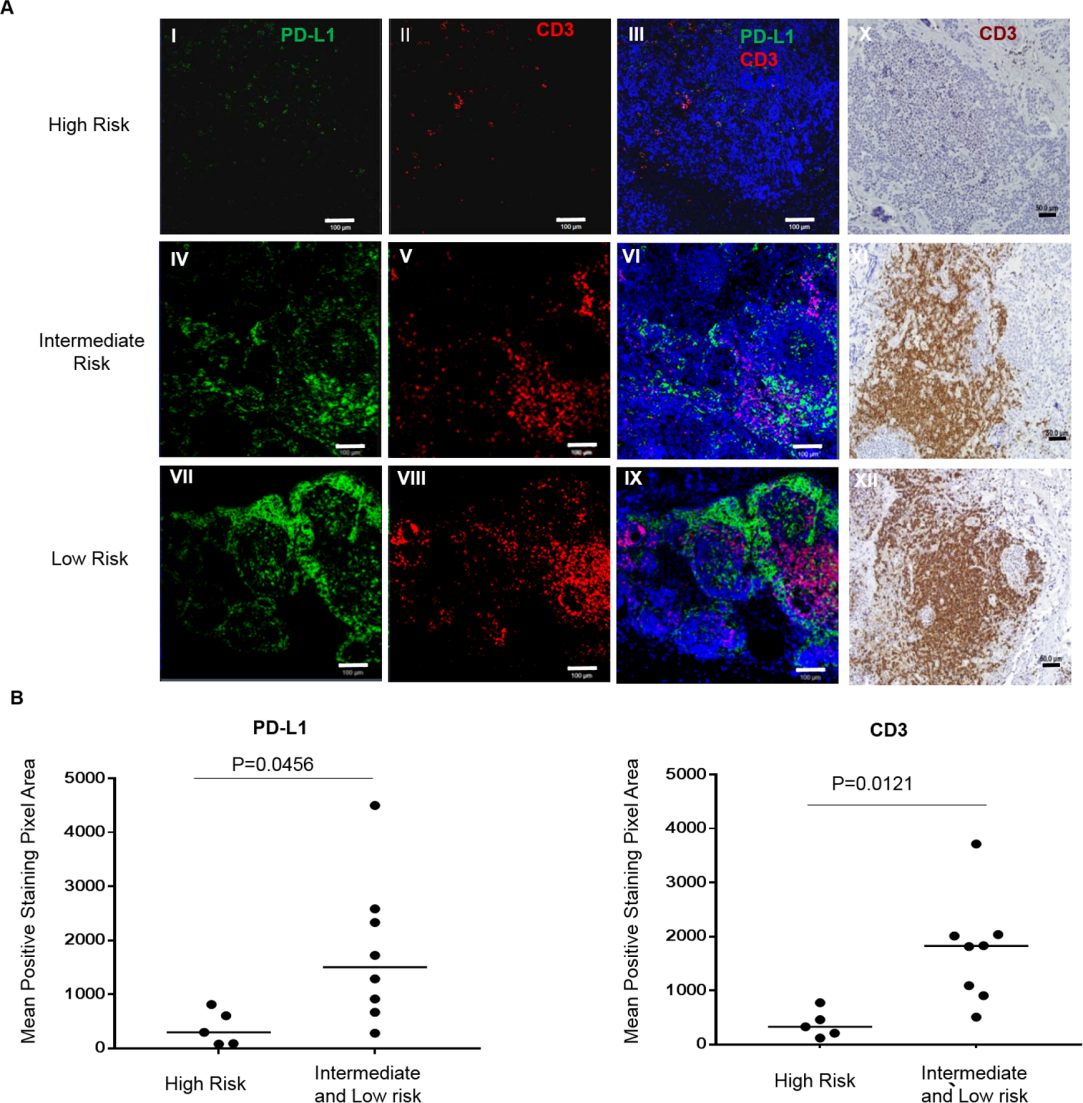
T-cell infiltrates and programmed cell death-ligand 1 (PD-L1) tumor expression are associated with risk stratification in human neuroblastoma. (A) Immunofluorescence (IF) double staining of PD-L1 (green) and CD3 (red) was performed on the paraffin-embedded neuroblastoma tumor tissue biopsied from high-risk (A I–III), intermediate-risk (A IV–VI), and low-risk (A VII–IX) tumors. The nuclei were stained with DAPI (blue), and the IF staining of CD3 was confirmed by immunohistochemical staining (X–XII). Tissue sections were imaged at 100× original magnification. The scale bar is 100 μm for (I–IX) and 50 μm for (X–XII). (B) The density of PD-L1 and CD3 staining as determined by digital image analysis shows significantly higher signal in low- and intermediate-risk groups compared to high-risk tumors. Each dot represents the mean fluorescent pixel area for a single subject. (*P*-values were calculated with an unpaired Student *t* test.).

PD-L1 and CD3 expression levels in various risk-stratified tumors were cataloged from newly diagnosed and untreated human specimens (Fig 6). The density of CD3^+^ TILs correlated with the expression of PD-L1 in human neuroblastoma tumor tissue. Dot plots showed that the distribution of CD3 and PD-L1 was statistically different between high- and intermediate/low-risk groups (Fig 6B). In general, human NB tumors of low (*n* = 3) and intermediate (*n* = 5) risk had high CD3^+^ TIL cell density and marked PD-L1 expression (Fig 6A IV–VI and VII–IX and 6B). In contrast, high-risk (*n* = 5) human tumors had very few CD3^+^ T-cell infiltrates and an absence of PD-L1 expression (Fig 6A I–III and 6B). These findings were similar to the mouse model in which immunogenic tumors had marked up-regulation of PD-L1 while the aggressive nonimmunogenic cell line (AgN2a) displayed minimal PD-L1 expression. These findings have implications for checkpoint immunotherapeutic strategies and also suggest that CD3^+^ TILs and PD-L1 expression could be useful prognostic indicators. Since PD-L1 is induced by T-cell activity, strong PD-L1 expression in the tumor reflects an immune-suppressive microenvironment against infiltrating T cells. Only the immunogenic low/intermediate-risk tumors exploit this protective mechanism, whereas high-risk tumors are nonimmunogenic, and thus, the PD-L1 pathway may be redundant. Taken together with the observed effects of vaccination in the mouse neuroblastoma models, PD-L1 blockade is necessary for effective vaccination against immunogenic tumors. High-risk nonimmunogenic tumors without TILs or PD-L1 expression will not be susceptible to checkpoint therapy alone but may be more susceptible to vaccination, if cell-mediated immunity can be induced against the tumor.

## Discussion

The work presented examines the role of adaptive immune resistance induced by PD-L1 in a mouse neuroblastoma model. Targeting PD-L1 enhanced the effectiveness of whole tumor cell vaccination when combined with CTLA-4 blockade. There is evidence in the literature suggesting that PD1 inhibition may be more effective than anti-CTLA-4 therapy [19], but our model and observations suggest that this may only be true for immunogenic tumors in which tumor-infiltrating T cells are already present but rendered incompetent through inhibition of the PD1/PD-L1 pathway. The use of combination checkpoint inhibition with vaccination proved more efficacious in this mouse neuroblastoma model, which is consistent with findings in the melanoma model [20]. Our findings show that CTLA-4 inhibition in the context of whole cell vaccination induced activation and expansion of TILs that were partially effective in controlling tumor growth. The TILs include both CD4^+^ and CD8^+^ subsets, but it is unclear whether CTLA-4 inhibition is acting directly on CD8 expansion or indirectly via CD4 helper function. It is also possible that CTLA-4 blockade may inactivate tumor-infiltrating T-reg [21], although our prior work in this model did not implicate T-reg infiltration following immune cell depletion studies [14]. Despite marked T-cell expansion and tumor infiltration following whole cell vaccination plus anti-CTLA-4 therapy alone, a significant proportion of tumors continued to grow (40%). The expression of PD-L1 on tumor cells induces adaptive tumor resistance. PD1 expressed on TILs is thought to be “exhausted” due to chronic stimulation by tumor antigens [22], yet in our tumor model, 80% of activated TILs expressed PD1. Despite this observation, blockade of PD-L1 did not change expression of these “exhaustion” markers on the T cells themselves but rendered TILs more effective in ablating tumor growth in all mice studied and in ex vivo cellular studies. The tumor cure rate was remarkable, and the combination of checkpoint inhibition may prove critical for tumor vaccine therapy of solid tumors.

Prior work from our laboratory showed that the aggressive nonimmunogenic mouse neuroblastoma (AgN2a) was surprisingly sensitive to Id2kd whole tumor cell vaccination and anti-CTLA-4 therapy alone [14]. Host immunity in this model was identical to the WT immunogenic Neuro2a tumor; thus, we hypothesized that the tumor’s lack of immune resistance was responsible for this enhanced sensitivity. On evaluating gene array analysis of nonimmunogenic AgN2a cells compared to the parent immunogenic Neuro2a cell line, we identified down-regulation of several tumor immunosuppressive pathways, including PD-L1 (3.6-fold), CD47 (3.3-fold), CD74 (6-fold), and CD40 (2.3-fold). This finding was unexpected, but the absence of these molecular pathways may speak to the lack of AgN2a tumor immunogenicity and thus redundancy for immune evasive tumor protective mechanisms. If true, this observation implies that nonimmunogenic tumors may be less resistant to host immunity if potent cellular immunity can be generated against the tumor. PD-L1 appears critical for generating both intrinsic and adaptive immune resistance in the wild-type Neuro2a tumor; thus, we focused on this axis in the AgN2a model. Baseline PD-L1 expression as well as IFNγ induction of PD-L1 in AgN2a was markedly reduced, as was expression in AgN2a tumors following vaccination, despite significant T-cell infiltrates. Taken together, these findings suggest that the lack of PD-L1 in AgN2a may enhance sensitivity to infiltrating TILs, which could have important implications for immunotherapy of nonimmunogenic high-risk disease. Under these conditions, the barrier to effective immune therapy in nonimmunogenic tumors would be induction of T-cell immunity. Our models suggest that whole tumor cell vaccination with Id2kd cells plus anti-CTLA-4 induces the appropriate T-cell response needed. These preclinical findings demonstrate that effective immunity can be generated against nonimmunogenic tumors and that vaccine therapy could be even more effective treatment as adaptive immune resistance seems to be of lesser significance.

Blockade of PD1/PD-L1 or CTLA-4 with other therapies has Food and Drug Administration (FDA) approval and is used against several tumor types [22–26]. Most monotherapies only achieve partial responses rather than complete responses. Combinations of checkpoint inhibitors may be more effective but are associated with more extensive adverse events when administered as nonspecific immune modulators [27]. A limitation of any immunotherapy is the potential to induce immunity against self and thus precipitate autoimmune disease or immune-related adverse events (irAEs). Therapy-induced irAEs are reported to be severe in 15%–30% of patients receiving anti-CTLA-4 alone and sometimes result in fatality [28]. Checkpoint inhibitors are frequently administered in multiple cycles until response or resistance is observed. As currently approved, the inhibitors are not given in the context of a vaccine, and if the patient has any propensity for autoimmunity, the checkpoint inhibitors may precipitate these effects. Also, blocking PD-L1 on the target tumor could hypothetically be of benefit by diminishing unwanted off-target effects, which could be less specific when blocking PD1 expression on circulating T cells. In our vaccine model, the combination of checkpoint inhibitors in the context of vaccine antigen, the relatively short exposure to immune modulators, and targeting inhibitory pathways on the tumor tissue itself may hypothetically contribute to fewer irAEs. All surviving mice were healthy and showed no signs of irAEs when followed for at least a year, although neither specific tissue biopsies nor serum markers were followed. Despite these positive findings, only a few doses of checkpoint inhibitors alone can induce profound dysregulation of immunity that may manifest as severe autoimmune events. Despite the lack of obvious irAEs, we also wished to determine if a short course of vaccine therapy (6 days) against established tumor would induce significant immune memory. Mice rechallenged with tumor cells as far out as 6 months following treatment retained immune memory and rejected the tumor cell rechallenge. In support of these survival observations, marked IFNγ secretion was detected from splenocytes harvested from vaccinated mice as late as 6 months following vaccination when cultured with WT tumor cells. In the context of therapy, these findings are promising for tumor vaccination in that unlike standard therapies, immunity against the tumor is preserved and may prevent recurrence of disease following complete response with improved event-free survival (EFS).

Immunohistochemistry and confocal microscopy of mouse and human tumors allowed for imaging of the inflammatory tumor microenvironment. The relevance of immunity in the mouse neuroblastoma model to human neuroblastoma was substantiated in our study by elucidating the interplay between host response and PD-L1 in the tumor microenvironment. Low- and intermediate-risk tumors biopsied prior to any therapy had the greatest number of T-cell infiltrates. Similar to the mouse tumor findings, PD-L1 was up-regulated and associated with CD3 T-cell expression, whereas high-risk tumors had very few T cells and minimal PD-L1 expression, similar to the nonimmunogenic AgN2a aggressive tumors. In several ways, these findings are confirmatory of recently published data from a large patient cohort. High CD3 infiltrates were noted in patients with good outcomes, while low CD3 infiltrates were associated with poor outcomes [29]. Furthermore, in this same publication, MYCN-amplified tumors lacked PD-L1 expression (3 of our 5 high-risk tumors were MYCN amplified); however, their findings also show that both absent PD-L1 expression and high PD-L1 expression were associated with subgroups of poor-acting tumors [29]. PD-L1 expression is of particular clinical interest in that reported studies of pretreatment PD-L1 tumor expression correlated with the likelihood of anti-PD1 response in patients [30, 31]. Thus, checkpoints alone in the low- and intermediate-risk inflammatory neuroblastoma tumors may be predictive of clinical response, whereas checkpoint inhibitors alone in high-risk tumors with minimal cell infiltrates will probably fail to have much clinical benefit.

Limitations of this study include the artificially induced mouse neuroblastoma model that may not replicate immunity in spontaneously occurring human neuroblastoma. Furthermore, mice were treated 6 days after inoculation with tumor cells, and large tumors established prior to initiating therapy will most likely behave differently because of mechanical properties that limit cellular immunity. The value of this model for testing the vaccine strategy, however, cannot be underemphasized in neuroblastoma, as the tumor is frequently reduced to minimal disease with standard therapies, but the recurrence rate is high, and any vaccine treatment would be best suited to use during the window of minimal residual disease. Finally, correlations are made between mouse and human models, but the number of both mouse and human correlates is limited.

**Fig 7:**
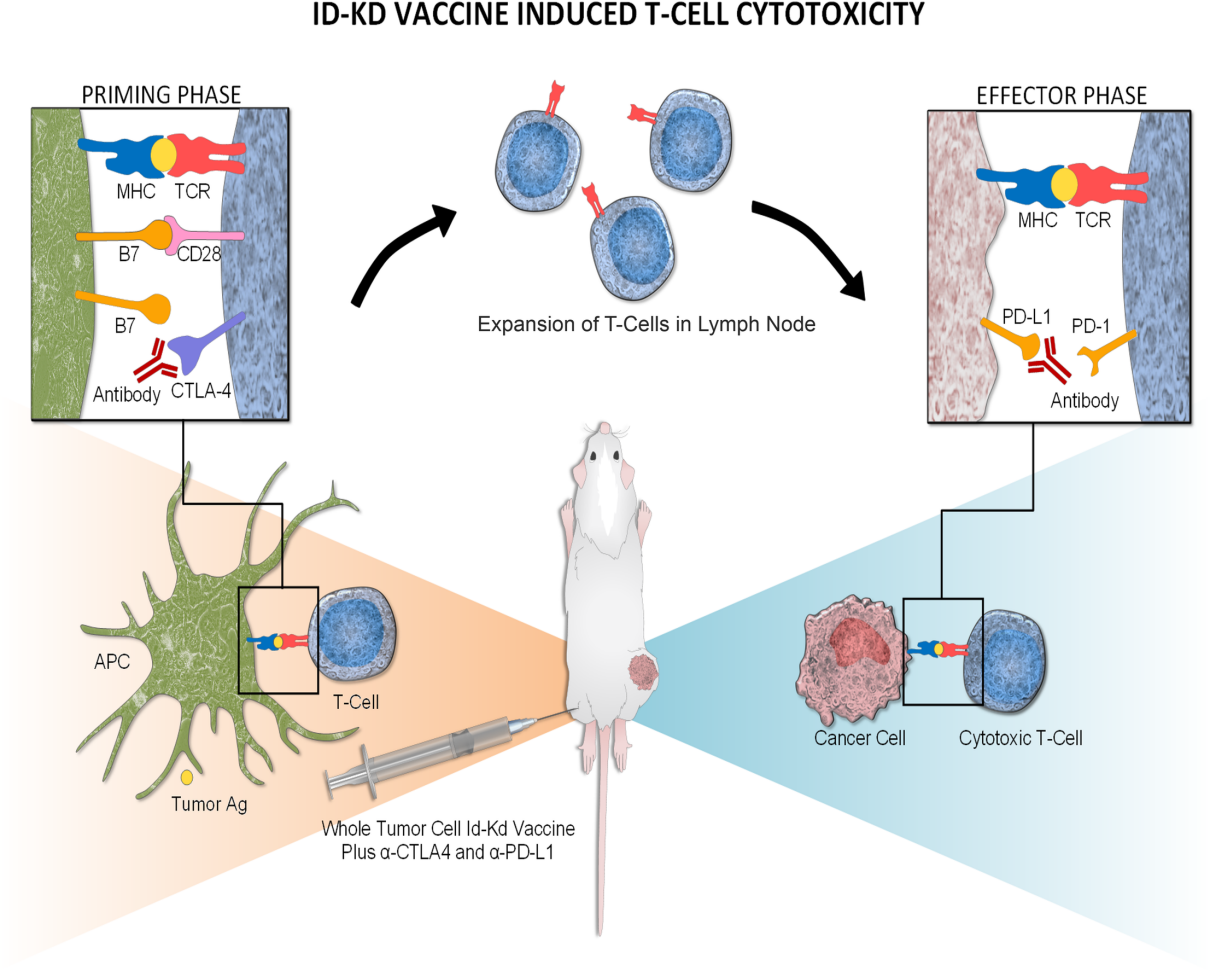
Mechanism of Id2kd Neuro2a vaccination combined with α-CTLA-4 and α-PD-L1 immunotherapy in the immunogenic Neuro2a model. During the priming phase, CTLA-4 blockade enhances the activation and proliferation of T cells that express programmed cell death 1 (PD1) and migrate to the tumor. Programmed cell death-ligand 1 (PD-L1) is up-regulated on the tumor cells, inducing adaptive resistance. Blockade of PD-L1 allows for enhanced cytotoxic effector function of the CD8^+^ tumor-infiltrating lymphocytes. In the nonimmunogenic model (AgN2a), adaptive resistance through PD-L1 is of less importance. APC, antigen-presenting cell; MHC, major histocompatibility complex; TCR, T-cell receptor. (Artwork generated by Olivia Abbate).

In conclusion, there are substantial advantages to combining checkpoint inhibitors with tumor vaccination in a model of neuroblastoma immunotherapy. Specifically, checkpoint blockade is administered in the context of tumor Ag, and thus, T-cell expansion is directed against tumor-specific antigens. CTLA-4 inhibition induces rapid proliferation and expansion of T cells, while PD-L1 blockade overcomes adaptive immune resistance on the tumor itself by enhancing the efficacy of effector T cells (Fig 7). The relatively short course of immune therapy and the targeted blockade of tumor suppressive signals resulted in minimal clinical irAEs in the mouse tumor model. Despite the apparent lack of irAEs, the amplified tumor-specific immune memory is potent, protective, and of long-term duration. These critical observations seem pertinent to human neuroblastoma, for which the mouse immunogenic and nonimmunogenic neuroblastoma models mimic the inflammatory microenvironment of low/intermediate- and high-risk disease, respectively.

## Supporting information

S1 Text Animal Research: Reporting of *In Vivo* Experiments (ARRIVE) checklist. (DOCX)

S1 Fig Fig 1A: Individual flow cytometry histogram plots for mouse neuroblastoma cell line wild-type (WT) N2a and human neuroblastoma cell lines SK-NSH and SY5Y with untreated control or 0.1, 1.0, and 10.0 ng/ml interferon gamma (IFNγ). (PPTX)

S2 Fig Fig 1C: Individual flow cytometry dot plots for expression of CD8, programmed cell death 1 (PD1), TIM3, and LAG3 on the surface of tumor-infiltrating lymphocytes isolated from diminishing tumors of mice treated with a-CTLA-4 and Id2kd whole cell vaccine. (PPTX)

S3 Fig Fig 4A: Individual flow cytometry histogram plots for the following groups in the cytotoxicity assay: Wild-type (WT) N2a+TIL, WT N2a+α-PD-L1+TIL, WT N2a+IFNγ+TIL, and WT N2a+IFNγ+α-PD-L1+TIL. N2a target cells are labeled with far-red dye. Killing of targets is detected by the shift in the combined far-red and caspase-positive population.
(PPTX)

S4 Fig Fig 5C: Individual flow cytometry histogram plots for mouse neuroblastoma cell line AgN2a control and treated with 0.1, 1.0, and 10.0 ng/ml interferon gamma (IFNγ). (PPTX)

S5 Fig Fig 5D: Individual flow cytometry histogram plots for human neuroblastoma cell line IMR32 control and treated with 0.1, 1.0, and 10.0 ng/ml interferon gamma (IFNγ). (PPTX)

S1 Data Fig 2C: Progress of tumor growth over time measured for individual mice in each of the following treatment groups: Wild-type (WT) N2a untreated control, WT N2a+ α-PD-L1, WT N2a+ α-PD-L1+Id2kd N2a, WT N2a+α-PD-L1+α-CTLA-4+Id2kd N2a, and WT N2a+ α-PD-L1+α-CTLA-4. Tumor volume was calculated using the following formula: (large diameter × small diameter)^2^ × 0.52.
(XLSX)

S2 Data Fig 2D: Average tumor growth for each treatment group represented in Fig 2C. The sum of tumor measurements in each group was divided by the number of mice. Averages were calculated until the first mouse in each group had to be euthanized. A Kaplan-Meier data chart for plotting the survival curve is shown.
(XLSX)

S3 Data Fig 3A: Enzyme-Linked ImmunoSpot (ELISpot) results for N2a cells treated with checkpoint blockers α-PD-L1, α-PD1, and α-TIM3 and cocultured with tumor-infiltrating lymphocytes (TILs) isolated from tumors of mice treated with vaccine and α-CTLA-4. The interferon gamma (IFNγ)-positive spots in each well were counted and graphed.
(XLSX)

S4 Data Fig 3B: Real-time quantitative PCR data (RT-qPCR) for PD-L1 expression of wild-type Neuro2a and aggressive Neuro2a (AgN2a). RT-qPCR of cell lines was performed in triplicate. Averages and standard error (SE) data are included.
(XLSX)

S5 Data Fig 4B: ELISA results to detect interferon gamma (IFNγ) produced from coculture of N2a and splenocytes of naïve mice as well as mice that were 6-month survivors of vaccine and dual checkpoint inhibitor therapy. IFNγ levels were undetectable in naïve mice.
(XLSX)

S6 Data Fig 5B: Expression levels of programmed cell death-ligand 1 (PD-L1) were measured using real-time quantitative PCR analysis using cDNA derived from total RNA from wild-type N2a cells and AgN2a cells. Expression levels were normalized to that of glyceraldehyde 3-phosphate dehydrogenase (GAPDH), calculated by the delta-delta Ct method and represented as a fold change over the expression in wild-type (WT) N2a. The expression of each gene was measured in triplicate wells, and standard error (SE) values have been indicated. The levels of the PD-L1 were reduced in AgN2a cells.
(XLSX)

S7 Data Fig 6B: 10–15 randomly selected fields in each stained human specimen were imaged under 100× magnification. Quantification of fluorescent intensity was achieved using Olympus cellSens imaging software (version 1.7). The fluorescent intensity in each field was measured. Data were presented as the mean fluorescent intensity of all the fields for each specimen. SE is standard error.
(XLSX)

## Abbreviations

EFS: event-free survival
ELISpot: Enzyme-Linked ImmunoSpot
IF: mmunofluorescence
FDA: Food and Drug Administration
IFNγ: interferon gamma
irAE: immune-related adverse event
PD1: programmed cell death 1
PD-L1: programmed cell death-ligand 1
RT-qPCR: real-time quantitative PCR
SE: standard error
TIL: tumor-infiltrating lymphocyte
WT: wild type

## References

[1] Brodeur GM, Bagatell R. Mechanisms of neuroblastoma regression. Nat Rev Clin Oncol 2014,11:704–713. doi: 10.1038/nrclinonc.2014.168 25331179 10.1038/nrclinonc.2014.168 PMC4244231

[2] Louis CU, Shohet JM. Neuroblastoma: molecular pathogenesis and therapy. Annu Rev Med 2015,66:49–63. doi: 10.1146/annurev-med-011514-023121 25386934 10.1146/annurev-med-011514-023121 PMC4418018

[3] Maris JM, Hogarty MD, Bagatell R, Cohn SL. Neuroblastoma. Lancet 2007,369:2106–2120. doi: 10.1016/S0140-6736(07)60983-0 17586306 10.1016/S0140-6736(07)60983-0

[4] Pardoll DM. The blockade of immune checkpoints in cancer immunotherapy. Nat Rev Cancer 2012,12:252–264. doi: 10.1038/nrc3239 22437870 10.1038/nrc3239 PMC4856023

[5] Schwartz RH. Costimulation of T lymphocytes: the role of CD28, CTLA-4, and B7/BB1 in interleukin-2 production and immunotherapy. Cell 1992,71:1065–1068. 1335362 10.1016/s0092-8674(05)80055-8

[6] Lenschow DJ, Walunas TL, Bluestone JA. CD28/B7 system of T cell costimulation. Annu Rev Immunol 1996,14:233–258. doi: 10.1146/annurev.immunol.14.1.233 8717514 10.1146/annurev.immunol.14.1.233

[7] Egen JG, Allison JP. Cytotoxic T lymphocyte antigen-4 accumulation in the immunological synapse is regulated by TCR signal strength. Immunity 2002,16:23–35. 11825563 10.1016/s1074-7613(01)00259-x

[8] Sfanos KS, Bruno TC, Meeker AK, De Marzo AM, Isaacs WB, Drake CG. Human prostate-infiltrating CD8+ T lymphocytes are oligoclonal and PD-1+. Prostate 2009,69:1694–1703. doi: 10.1002/pros.21020 19670224 10.1002/pros.21020 PMC2782577

[9] Ahmadzadeh M, Johnson LA, Heemskerk B, Wunderlich JR, Dudley ME, White DE, et al Tumor antigen-specific CD8 T cells infiltrating the tumor express high levels of PD-1 and are functionally impaired. Blood 2009,114:1537–1544. doi: 10.1182/blood-2008-12-195792 19423728 10.1182/blood-2008-12-195792 PMC2927090

[10] Ishida Y, Agata Y, Shibahara K, Honjo T. Induced expression of PD-1, a novel member of the immunoglobulin gene superfamily, upon programmed cell death. EMBO J 1992,11:3887–3895. 1396582 10.1002/j.1460-2075.1992.tb05481.x PMC556898

[11] Freeman GJ, Long AJ, Iwai Y, Bourque K, Chernova T, Nishimura H, et al Engagement of the PD-1 immunoinhibitory receptor by a novel B7 family member leads to negative regulation of lymphocyte activation. J Exp Med 2000,192:1027–1034. 11015443 10.1084/jem.192.7.1027 PMC2193311

[12] Keir ME, Butte MJ, Freeman GJ, Sharpe AH. PD-1 and its ligands in tolerance and immunity. Annu Rev Immunol 2008,26:677–704. doi: 10.1146/annurev.immunol.26.021607.090331 18173375 10.1146/annurev.immunol.26.021607.090331 PMC10637733

[13] Chakrabarti L, Wang BD, Lee NH, Sandler AD. A mechanism linking Id2-TGFbeta crosstalk to reversible adaptive plasticity in neuroblastoma. PLoS ONE 2013,8:e83521 doi: 10.1371/journal.pone.0083521 24376712 10.1371/journal.pone.0083521 PMC3871549

[14] Chakrabarti L, Morgan C, Sandler AD. Combination of Id2 Knockdown Whole Tumor Cells and Checkpoint Blockade: A Potent Vaccine Strategy in a Mouse Neuroblastoma Model. PLoS ONE 2015,10:e0129237 doi: 10.1371/journal.pone.0129237 26079374 10.1371/journal.pone.0129237 PMC4469424

[15] He L, Hakimi J, Salha D, Miron I, Dunn P, Radvanyi L. A sensitive flow cytometry-based cytotoxic T-lymphocyte assay through detection of cleaved caspase 3 in target cells. J Immunol Methods 2005,304:43–59. doi: 10.1016/j.jim.2005.06.005 16076473 10.1016/j.jim.2005.06.005

[16] Zitvogel L, Kroemer G. Targeting PD-1/PD-L1 interactions for cancer immunotherapy. Oncoimmunology 2012,1:1223–1225. doi: 10.4161/onci.21335 23243584 10.4161/onci.21335 PMC3518493

[17] Gros A, Robbins PF, Yao X, Li YF, Turcotte S, Tran E, et al PD-1 identifies the patient-specific CD8(+) tumor-reactive repertoire infiltrating human tumors. J Clin Invest 2014,124:2246–2259. doi: 10.1172/JCI73639 24667641 10.1172/JCI73639 PMC4001555

[18] Schwab M, Ellison J, Busch M, Rosenau W, Varmus HE, Bishop JM. Enhanced expression of the human gene N-myc consequent to amplification of DNA may contribute to malignant progression of neuroblastoma. Proc Natl Acad Sci U S A 1984,81:4940–4944. 6589638 10.1073/pnas.81.15.4940 PMC391608

[19] Parry RV, Chemnitz JM, Frauwirth KA, Lanfranco AR, Braunstein I, Kobayashi SV, et al CTLA-4 and PD-1 receptors inhibit T-cell activation by distinct mechanisms. Mol Cell Biol 2005,25:9543–9553. doi: 10.1128/MCB.25.21.9543-9553.2005 16227604 10.1128/MCB.25.21.9543-9553.2005 PMC1265804

[20] Curran MA, Montalvo W, Yagita H, Allison JP. PD-1 and CTLA-4 combination blockade expands infiltrating T cells and reduces regulatory T and myeloid cells within B16 melanoma tumors. Proc Natl Acad Sci U S A 2010,107:4275–4280. doi: 10.1073/pnas.0915174107 20160101 10.1073/pnas.0915174107 PMC2840093

[21] Spranger S, Koblish HK, Horton B, Scherle PA, Newton R, Gajewski TF. Mechanism of tumor rejection with doublets of CTLA-4, PD-1/PD-L1, or IDO blockade involves restored IL-2 production and proliferation of CD8(+) T cells directly within the tumor microenvironment. J Immunother Cancer 2014,2:3 doi: 10.1186/2051-1426-2-3 24829760 10.1186/2051-1426-2-3 PMC4019906

[22] Barber DL, Wherry EJ, Masopust D, Zhu B, Allison JP, Sharpe AH, et al Restoring function in exhausted CD8 T cells during chronic viral infection. Nature 2006,439:682–687. doi: 10.1038/nature04444 16382236 10.1038/nature04444

[23] Butte MJ, Keir ME, Phamduy TB, Sharpe AH, Freeman GJ. Programmed death-1 ligand 1 interacts specifically with the B7-1 costimulatory molecule to inhibit T cell responses. Immunity 2007,27:111–122. doi: 10.1016/j.immuni.2007.05.016 17629517 10.1016/j.immuni.2007.05.016 PMC2707944

[24] Okazaki T, Chikuma S, Iwai Y, Fagarasan S, Honjo T. A rheostat for immune responses: the unique properties of PD-1 and their advantages for clinical application. Nat Immunol 2013,14:1212–1218. doi: 10.1038/ni.2762 24240160 10.1038/ni.2762

[25] Tumeh PC, Harview CL, Yearley JH, Shintaku IP, Taylor EJ, Robert L, et al PD-1 blockade induces responses by inhibiting adaptive immune resistance. Nature 2014,515:568–571. doi: 10.1038/nature13954 25428505 10.1038/nature13954 PMC4246418

[26] Mahoney KM, Freeman GJ, McDermott DF. The Next Immune-Checkpoint Inhibitors: PD-1/PD-L1 Blockade in Melanoma. Clin Ther 2015,37:764–782. doi: 10.1016/j.clinthera.2015.02.018 25823918 10.1016/j.clinthera.2015.02.018 PMC4497957

[27] Wolchok JD, Kluger H, Callahan MK, Postow MA, Rizvi NA, Lesokhin AM, et al Nivolumab plus ipilimumab in advanced melanoma. N Engl J Med 2013,369:122–133. doi: 10.1056/NEJMoa1302369 23724867 10.1056/NEJMoa1302369 PMC5698004

[28] Topalian SL, Taube JM, Anders RA, Pardoll DM. Mechanism-driven biomarkers to guide immune checkpoint blockade in cancer therapy. Nat Rev Cancer 2016,16:275–287. doi: 10.1038/nrc.2016.36 27079802 10.1038/nrc.2016.36 PMC5381938

[29] Melaiu O, Mina M, Chierici M, Boldrini R, Jurman G, Romania P, et al PD-L1 Is a Therapeutic Target of the Bromodomain Inhibitor JQ1 and, Combined with HLA Class I, a Promising Prognostic Biomarker in Neuroblastoma. Clin Cancer Res 2017. 10.1158/1078-0432.CCR-16-2601 28270499

[30] Brahmer JR, Drake CG, Wollner I, Powderly JD, Picus J, Sharfman WH, et al Phase I study of single-agent anti-programmed death-1 (MDX-1106) in refractory solid tumors: safety, clinical activity, pharmacodynamics, and immunologic correlates. J Clin Oncol 2010,28:3167–3175. doi: 10.1200/JCO.2009.26.7609 20516446 10.1200/JCO.2009.26.7609 PMC4834717

[31] Topalian SL, Hodi FS, Brahmer JR, Gettinger SN, Smith DC, McDermott DF, et al Safety, activity, and immune correlates of anti-PD-1 antibody in cancer. N Engl J Med 2012,366:2443–2454. doi: 10.1056/NEJMoa1200690 22658127 10.1056/NEJMoa1200690 PMC3544539

