# Supplementary material for: PD-L1 checkpoint inhibition and anti-CTLA-4 whole tumor cell vaccination counter adaptive immune resistance: A mouse neuroblastoma model that mimics human disease
Source: PLoS Med. 2018 Jan 29;15(1):e1002497. doi: 10.1371/journal.pmed.1002497 (PMC5788338; doi:10.1371/journal.pmed.1002497)
Supplement: S1 Text — (DOCX) [file pmed.1002497.s001.docx]

**The ARRIVE Checklist**

**TITLE**

**1 Provide as accurate and concise a description of the content of the article as possible.**

**PD-L1 checkpoint inhibition and anti-CTLA4 whole tumor cell vaccination counters adaptive immune resistance: a mouse neuroblastoma model that mimics human disease**

**ABSTRACT**

**2 Provide an accurate summary of the background, research objectives (including details of the species or strain of animal used), key methods, principal findings, and conclusions of the study.**

**Background**

Adaptive immune resistance induces an immunosuppressive tumor environment that enables immune evasion. This phenomenon results in tumor escape with progression and metastasis. Programmed cell death-ligand 1 (PD-L1) expressed on tumors is thought to inhibit tumor infiltrating lymphocytes (TIL) through programmed cell death 1 (PD1), enabling adaptive immune resistance. This study investigates the role of PD-L1 in both mouse and human neuroblastoma immunity. The consequence of PD-L1 inhibition is characterized in the context of an established whole tumor cell vaccine.

**Methods and Findings**

A mouse model of neuroblastoma was investigated using an Id2-knock down whole cell vaccine in combination with checkpoint inhibition. We show that immunogenic mouse neuroblastoma acquires adaptive immune resistance by up-regulating PD-L1 expression, whereas PD-L1 is of lesser consequence in non-immunogenic neuroblastoma tumors. Combining PD-L1 checkpoint inhibition with whole tumor cell / anti-CTLA4 vaccination enhanced tumor cell killing, cured mice with established tumors and induced long-term immune memory (6 months). From an evaluation of patient neuroblastoma tumors, we found that the inflammatory environment of the mouse neuroblastoma mimicked human disease in which PD-L1 expression was associated directly with TIL and lower risk tumors. High-risk patient tumors were lacking both TIL and PD-L1 expression. Although a correlation in immunity seems to exist between the mouse model and human findings, the mouse tumor model is induced and not spontaneously occurring and furthermore, the number of both mouse and human correlates are limited.

**Conclusions**

This study demonstrates the role PD-L1 plays in neuroblastoma’s resistance to immunity and defines the non-redundant effect of combination checkpoint inhibition with vaccine therapy in a mouse model. High risk, non-immunogenic human tumors display both diminished PD-L1 expression and adaptive immune resistance. Paradoxically, high-risk tumors may be more responsive to effective vaccine therapy due to their apparent lack of adaptive immune resistance.

**INTRODUCTION**

**Background**

**3 a. Include sufficient scientific background (including relevant references to previous work) to understand the motivation and context for the study, and explain the experimental approach and rationale.**

**b. Explain how and why the animal species and model being used can address the scientific objectives and, where appropriate, the study’s relevance to human biology.**

1. The AJ mouse model is used as it is the syngeneic host for the murine Neuro2a neuroblastoma cell line. We previously showed that the combination of a whole cell vaccine combined with CTLA-4 blockade induces robust immunity with a 60% cure rate when the vaccine is given 6 days after Neuro2a challenge (Chakrabarti L, Morgan C, Sandler AD. Combination of Id2 Knockdown Whole Tumor Cells and Checkpoint Blockade: A Potent Vaccine Strategy in a Mouse Neuroblastoma Model. PloS one. 2015;10(6):e0129237. Epub 2015/06/17). The new work investigates the participation of another checkpoint (PD-L1) frequently expressed on tumors that is considered to inhibit tumor infiltrating lymphocytes. Our model using the immunocompetent host to demonstrate the immunologic changes that occur in the tumor itself following vaccination. Infiltrating T-cells and tumors are investigated for changes in the PD1:PD-L1 axis to evaluate potential adaptive immune resistance in the tumor following effective therapeutic vaccination. The findings present the rationale for combining checkpoint inhibitors in whole cell vaccination. T-cell infiltrates and PD-L1 expression are also investigated and compared to tumors from a non-immunogenic Neuro2a call line (AgN2a).
2. The immunogenic changes identified in the mouse model are then extended to an investigation of human neuroblastoma tumors. The findings in mice of T-cell infiltrates associated with PD-L1 tumor expression are also observed in the low and intermediate risk human neuroblastoma tumors. However, similar to the non-immunogenic Neuro2a mouse tumors, high risk human tumors lack both T-cell infiltrates and PD-L1 expression.

**Objectives**

**4 Clearly describe the primary and any secondary objectives of the study, or specific hypotheses being tested.**

- Primary objective: To investigate the role of PD-L1 and adaptive immune resistance in neuroblastoma vaccination and to determine whether combination checkpoint inhibition is advantageous
- Secondary objective: To evaluate whether cellular infiltrates and PD-L1 expression in human neuroblastoma correlates with observations in the mouse model

**METHODS**

**Ethical statement**

**5 Indicate the nature of the ethical review permissions, relevant licenses (e.g. Animal [Scientific Procedures] Act 1986), and national or institutional guidelines for the care and use of animals, that cover the research.**

These studies were carried out in accordance with the NIH Guide for the Care and Use of Laboratory Animals under Children’s National Medical Center IACUC protocol 30499.

**Study design**

**6 For each experiment, give brief details of the study design, including:**

**a. The number of experimental and control groups.**

**b. Any steps taken to minimise the effects of subjective bias when allocating animals to treatment (e.g., randomisation procedure) and when assessing results (e.g., if done, describe who was blinded and when).**

**c. The experimental unit (e.g. a single animal, group, or cage of animals).**

**A time-line diagram or flow chart can be useful to illustrate how complex study designs were carried out.**

a. At least 5 mice were used in each experimental group of mice that received N2a tumor cells with or without treatment. In the key complete vaccination experimental group 10 mice were used to expand the observation. (A prior single group of 6 mice was also treated with the full vaccination, however tumors were induced with chemiluminescent N2a so that the shrinking tumors could be visualized). Hence the total number of mice receiving both checkpoint inhibitors in combination with Id2 knockdown vaccine is n=16.

b. All the mice in a given experiment were inoculated with 1X10^6^ N2a cells to induce growth of tumors. Before starting treatment all inoculated mice were randomly assigned to different groups before baseline tumor measurements were taken, in order to reduce bias.

c. Tumor sizes on each mouse were calculated individually in order to generate tumor growth curves. To calculate average tumor growth all mice in a group were taken as a unit.

**Experimental procedures**

**7 For each experiment and each experimental group, including controls, provide precise details of all procedures carried out. For example:**

**a. How (e.g., drug formulation and dose, site and route of administration, anaesthesia and analgesia used [including monitoring], surgical procedure, method of euthanasia). Provide details of any specialist equipment used, including supplier(s).**

**b. When (e.g., time of day).**

**c. Where (e.g., home cage, laboratory, water maze).**

**d. Why (e.g., rationale for choice of specific anaesthetic, route of administration, drug dose used).**

N2a tumor cells and whole-cell Id2 knockdown N2a vaccine were administered subcutaneously to the flank of mice, based on our prior work. Checkpoint inhibitor antibodies were given intraperitoneally, according to established procedures. For in vivo imaging, mice were maintained under Isoflurane anesthesia, again following established protocols. Mice were humanely euthanized with CO2 gas followed by cervical dislocation according to our animal care protocol. All procedures involving mice were carried out in the Children’s National Medical Center research animal facility.

**Experimental animals**

**8 a. Provide details of the animals used, including species, strain, sex, developmental stage (e.g., mean or median age plus age range), and weight (e.g., mean or median weight plus weight range).**

**b. Provide further relevant information such as the source of animals, international strain nomenclature, genetic modification status (e.g. knock-out or transgenic), genotype, health/immune status, drug- or test naıve, previous procedures, etc.**

All mice used in this study were AJ mice, obtained from Jackson Laboratories, Maine. Female mice age 6 weeks were used, in the range of 18-20 gram weight. The details of the mice are listed in this link:

<https://www.jax.org/strain/000646>

**Housing and husbandry**

**9 Provide details of:**

**a. Housing (e.g., type of facility, e.g., specific pathogen free (SPF); type of cage or housing; bedding material; number of cage companions; tank shape and material etc. for fish).**

**b. Husbandry conditions (e.g., breeding programme, light/dark cycle, temperature, quality of water etc. for fish, type of food, access to food and water, environmental enrichment).**

**c. Welfare-related assessments and interventions that were carried out before, during, or after the experiment.**

1. Housing- conventional facility, housed at maximum 5 to a cage, ventilated cages, corncob bedding
2. Husbandry conditions: light/dark cycle (12hrs/12hrs) 7am-7pm; temperature 70^o^F ± 2 ^o^F; quality of water- acidified; environmental enrichment- shredded paper strips; food- Harlan Teklad standard pellet diet, food and water provided freely in cage
3. Not applicable

**Sample size**

**10 a. Specify the total number of animals used in each experiment and the number of animals in each experimental group.**

**b. Explain how the number of animals was decided. Provide details of any sample size calculation used.**

**c. Indicate the number of independent replications of each experiment, if relevant.**

The total number of mice used in the tumor growth studies is 36, and the number of animals in each experimental group was either 5,6 or 10. These group sizes have been used routinely in our lab and are deemed sufficient to obtain statistical analysis.

**Allocating animals to experimental groups**

**11 a. Give full details of how animals were allocated to experimental groups, including randomisation or matching if done.**

**b. Describe the order in which the animals in the different experimental groups were treated and assessed.**

All mice in a given experiment were inoculated with tumor on the same date, and then randomly assigned to different groups prior to the start of treatments. Animals in different groups were treated on the same schedule with different therapies**.**

**Experimental outcomes**

**12 Clearly define the primary and secondary experimental outcomes assessed (e.g., cell death, molecular markers, behavioural changes).**

Experimental outcomes were measured by comparing rate of tumor regression in different treatment groups.

**Statistical methods**

**13 a. Provide details of the statistical methods used for each analysis.**

**b. Specify the unit of analysis for each dataset (e.g. single animal, group of animals, single neuron).**

**c. Describe any methods used to assess whether the data met the assumptions of the statistical approach.**

For each statistical analysis, appropriate tests were selected on the basis of whether the data was normally distributed by using the D'Agostino & Pearson normality test. Data were analyzed using an unpaired two-tailed Student’s t test or an unpaired Mann-Whitney test for comparisons between two groups, and two-way repeated-measures ANOVA for behavioral tests.

Survival curves of mice were calculated according to the Kaplan-Meier method; survival analyses was performed using the logrank test. Statistical calculations were performed using GraphPad Prism software (GraphPad Software, San Diego, California) and the probability level of p<0.05 was considered significant.

**RESULTS**

**Baseline data
14 For each experimental group, report relevant characteristics and health status of animals (e.g., weight, microbiological status, and drug- or test-naıve) before treatment or testing (this information can often be tabulated).**

All animals were in normal health at the start of each study and were acclimated to the living conditions for one week prior to intervention.

**Numbers analyzed**

**15 a. Report the number of animals in each group included in each analysis. Report absolute numbers (e.g. 10/20, not 50%).**

**b. If any animals or data were not included in the analysis, explain why.**

Data provided in #6.

**Outcomes and estimation**

**16 Report the results for each analysis carried out, with a measure of precision (e.g., standard error or confidence interval).**

P < 0.03 was obtained for the group receiving both checkpoint inhibitors and Id2 knockdown vaccine as compared to other treatment groups.

**Adverse events**

**17 a. Give details of all important adverse events in each experimental group.**

**b. Describe any modifications to the experimental protocols made to reduce adverse events.**

No adverse events occurred during the course of these experiments.

**DISCUSSION**

**Interpretation/scientific implications**

**18 a. Interpret the results, taking into account the study objectives and hypotheses, current theory, and other relevant studies in the literature.**

**b. Comment on the study limitations including any potential sources of bias, any limitations of the animal model, and the imprecision associated with the results.**

**c. Describe any implications of your experimental methods or findings for the replacement, refinement, or reduction (the 3Rs) of the use of animals in research.**

The study shows that tumor bearing mice that received whole tumor cell vaccine and both anti-PDL1 and anti-CTLA4 antibodies achieved complete tumor regression when treatment is started 6 days following tumor cell inoculation. While we hypothesize that this finding may be applicable to human patients we are working with a mouse model that is artificial in that it is induced and not spontaneously occurring. Thus, a limitation of this induced mouse neuroblastoma model is that it may not replicate immunity in spontaneously occurring human neuroblastoma. Furthermore, mice were treated at 6 days after inoculating with tumor cells and large tumors established prior to initiating therapy will most likely behave differently due to mechanical properties that limit cellular immunity. The value of this model for testing the vaccine strategy however, cannot be under-emphasized in neuroblastoma as the tumor is frequently reduced to minimal disease with standard therapies, but the recurrence rate is high and any vaccine treatment would be best suited during the window of minimal residual disease.

**Generalisability/translation**

**19 Comment on whether, and how, the findings of this study are likely to translate to other species or systems, including any relevance to human biology.**

We have used the AJ model of induced mouse neuroblastoma to demonstrate the induction of tumor immunogenicity by down modulation of Id2 gene expression and combination checkpoint inhibition. This study serves as “proof of principle” in that robust non redundant immunity can be induced using whole cell vaccination in a tumor model. Since this response requires a functional host immune system we cannot use human neuroblastoma cell lines in a SCID or nude mouse model. Furthermore, the study of cellular immunity in the tumor micro-environment is of particular interest as the mouse model mimicked the human findings in patients. Immunogenic tumors were noticeable for robust T-cell infiltrates and PD-L1 expression, while non-immunogenic tumors lacked both T-cells and PD-L1 expression.

**Funding**

**20 List all funding sources (including grant number) and the role of the funder(s) in the study.**

This work has been supported in part by the EVAN and Catherine Blaire foundations and the Michael Sandler Cancer Research Fund as well as the Sheikh Zayed Institute for Pediatric Surgical Innovation at the Children’s National Medical Center. The funders had no role in any aspect of this study. This includes study design, data collection and analysis, decision to publish, or preparation of the manuscript.
