# Supplementary material for: PD-L1 checkpoint inhibition and anti-CTLA-4 whole tumor cell vaccination counter adaptive immune resistance: A mouse neuroblastoma model that mimics human disease
Source: PLoS Med. 2018 Jan 29;15(1):e1002497. doi: 10.1371/journal.pmed.1002497 (PMC5788338; doi:10.1371/journal.pmed.1002497)
Supplement: S1 Fig — (PPTX) [file pmed.1002497.s002.pptx]

## Slide 1
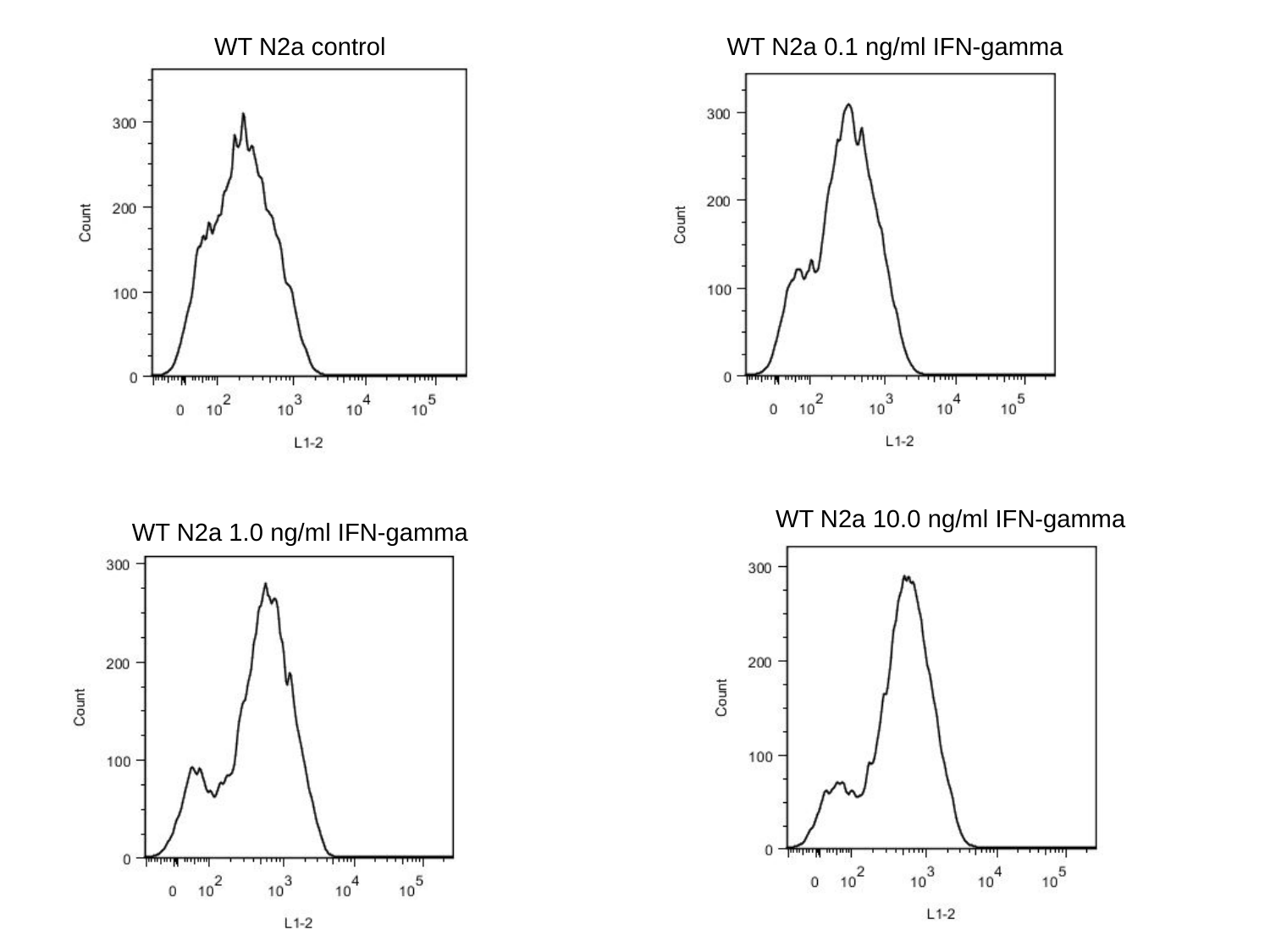

WT N2a control
WT N2a 0.1 ng/ml IFN-gamma
WT N2a 10.0 ng/ml IFN-gamma
WT N2a 1.0 ng/ml IFN-gamma

## Slide 2
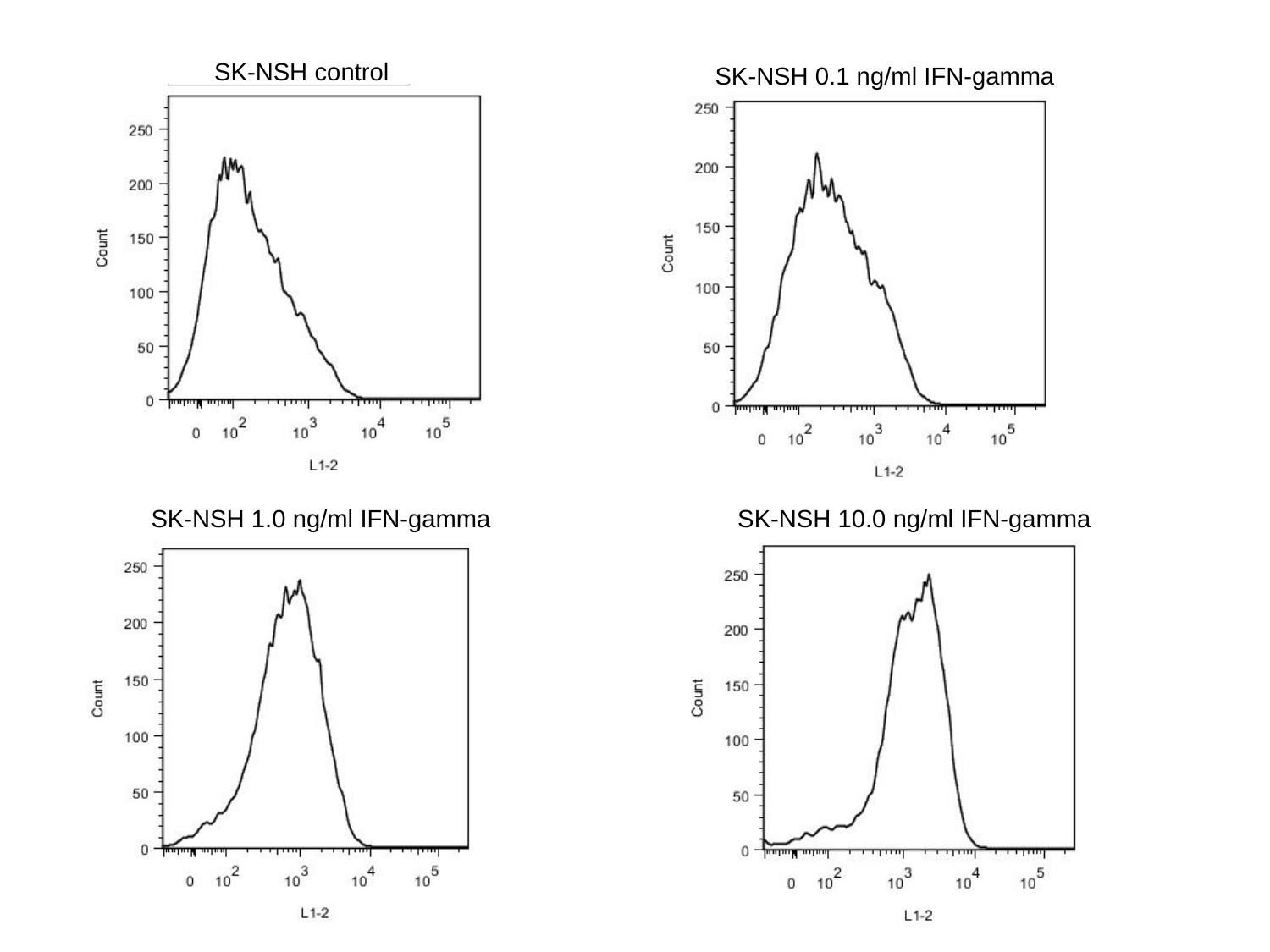

SK-NSH control
SK-NSH 0.1 ng/ml IFN-gamma
SK-NSH 1.0 ng/ml IFN-gamma
SK-NSH 10.0 ng/ml IFN-gamma

## Slide 3
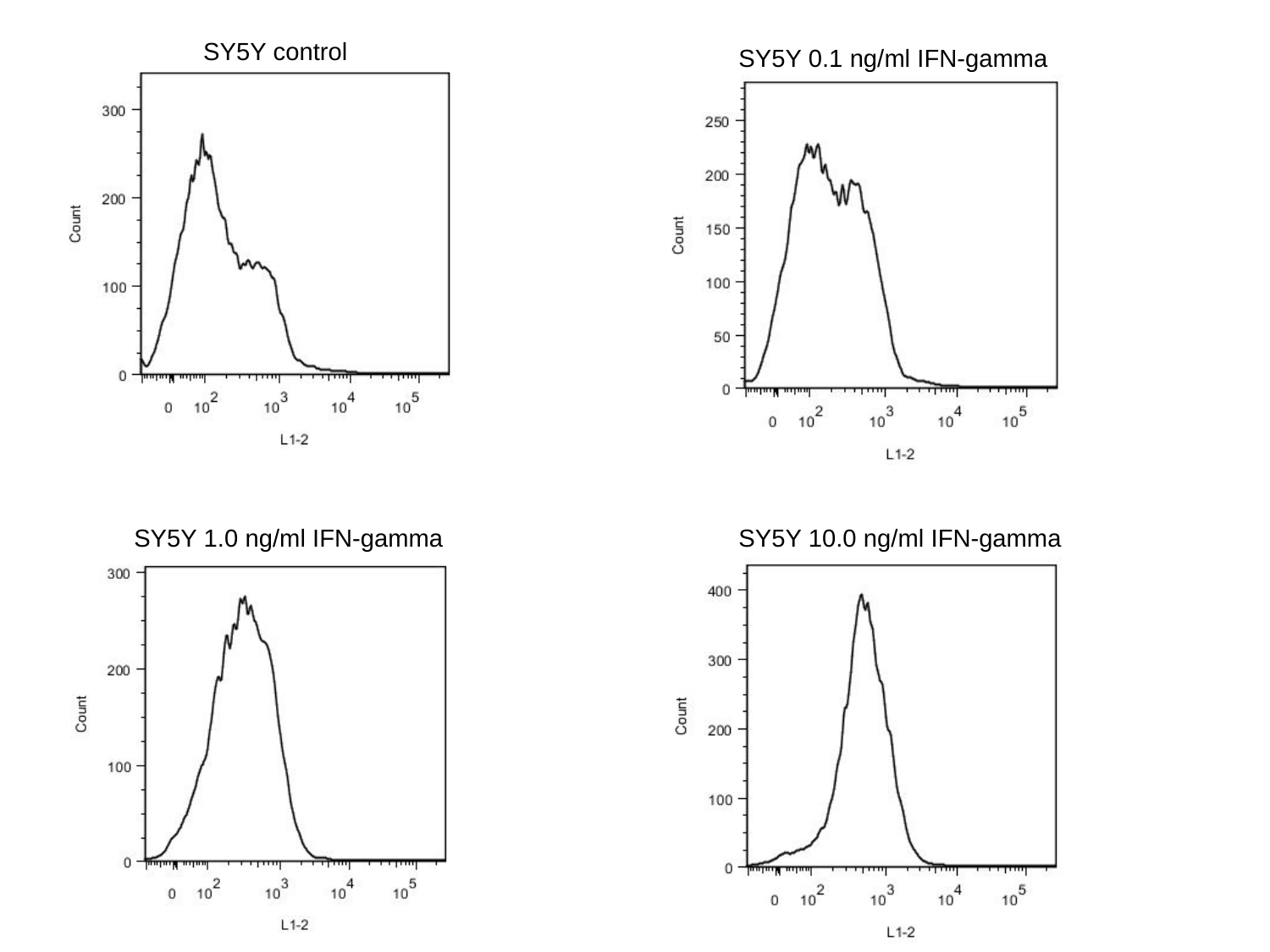

SY5Y control
SY5Y 0.1 ng/ml IFN-gamma
SY5Y 1.0 ng/ml IFN-gamma
SY5Y 10.0 ng/ml IFN-gamma
