# Supplementary figures and images for: PD-L1 checkpoint inhibition and anti-CTLA-4 whole tumor cell vaccination counter adaptive immune resistance: A mouse neuroblastoma model that mimics human disease
Source: PLoS Med. 2018 Jan 29;15(1):e1002497. doi: 10.1371/journal.pmed.1002497 (PMC5788338; doi:10.1371/journal.pmed.1002497)

## Slide 1
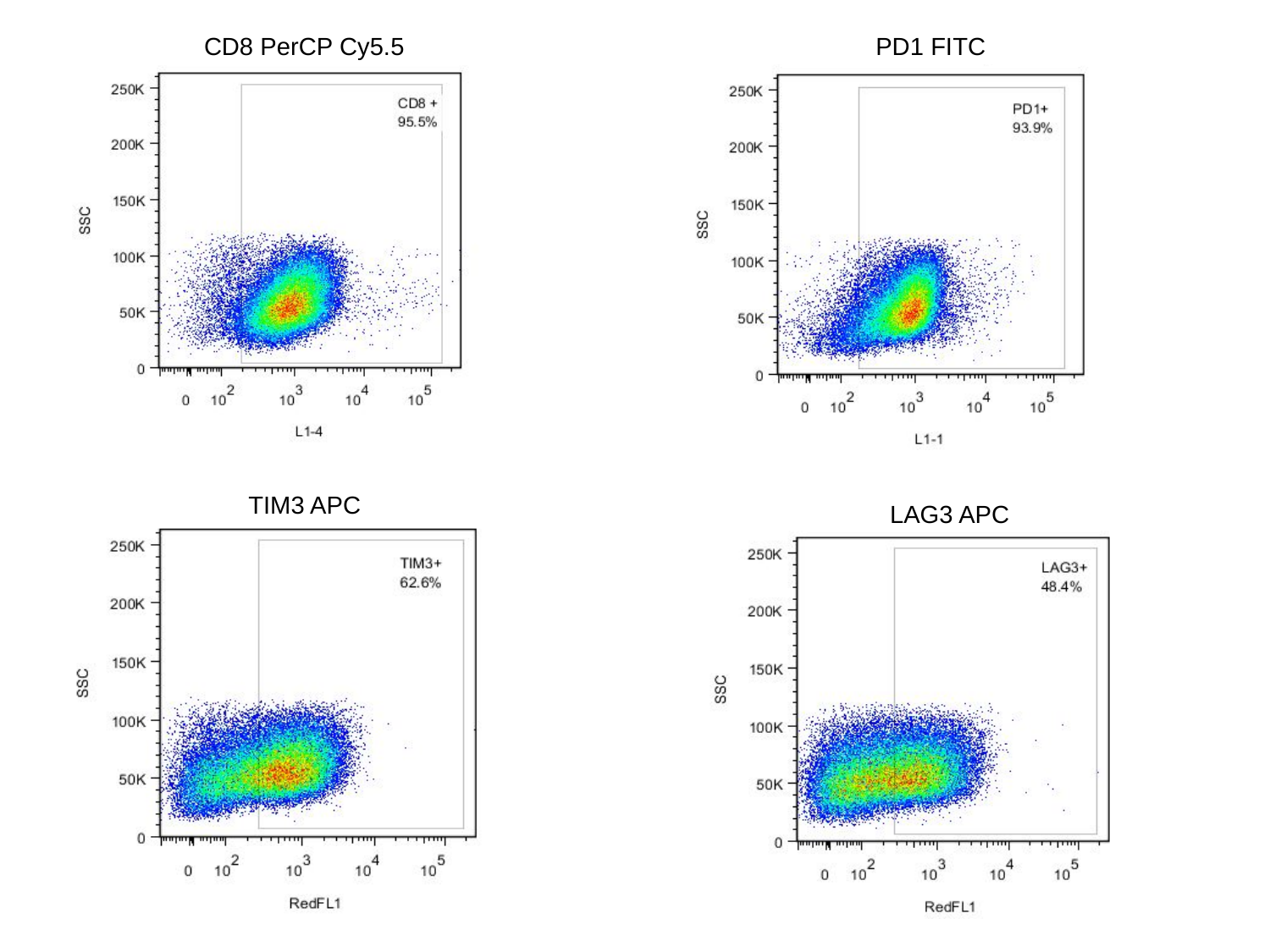

CD8 PerCP Cy5.5
PD1 FITC
TIM3 APC
LAG3 APC

Supplement: S2 Fig — (PPTX) [file pmed.1002497.s003.pptx]

## Slide 1
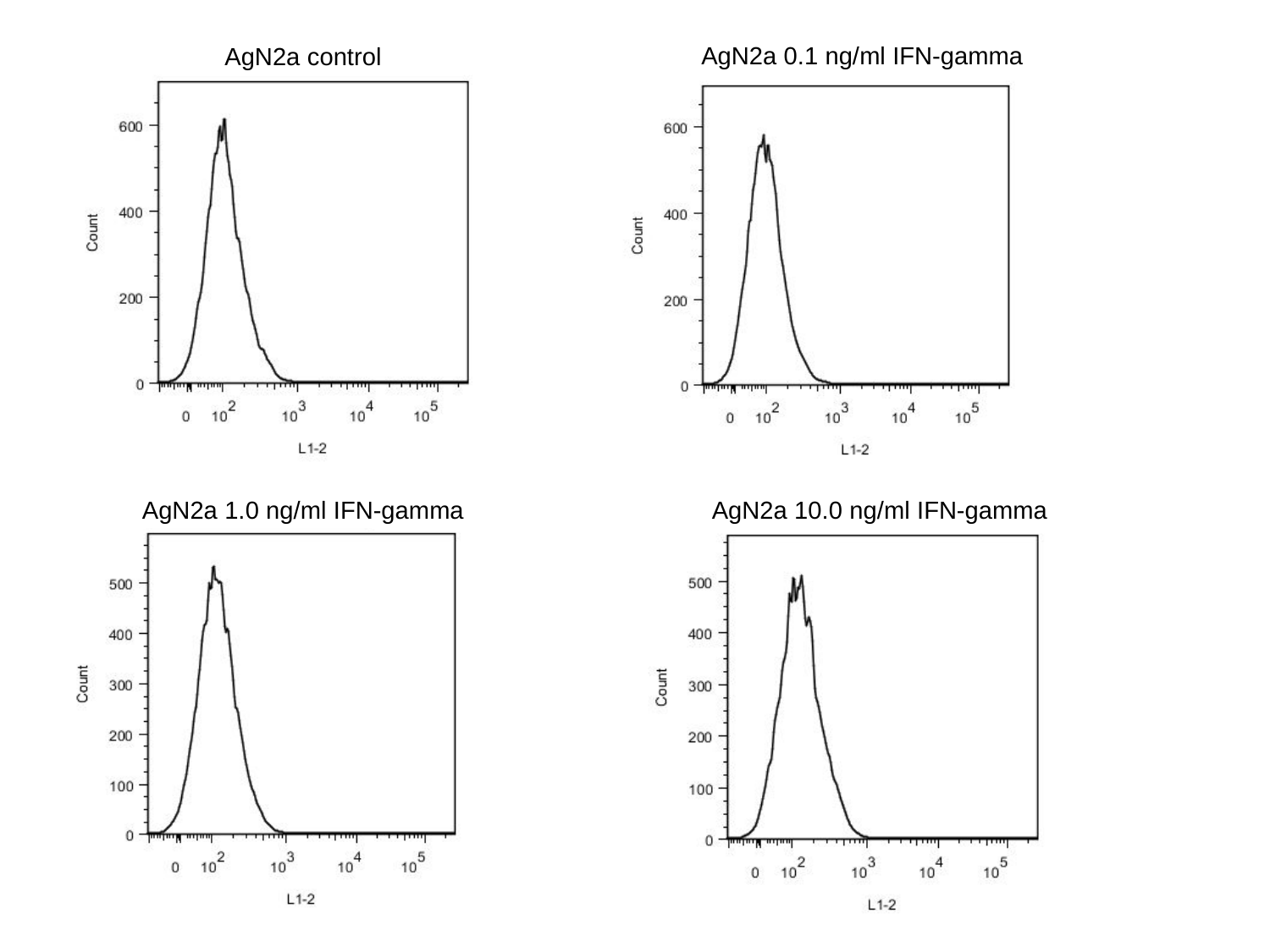

AgN2a 0.1 ng/ml IFN-gamma
AgN2a control
AgN2a 1.0 ng/ml IFN-gamma
AgN2a 10.0 ng/ml IFN-gamma

Supplement: S4 Fig — (PPTX) [file pmed.1002497.s005.pptx]

## Slide 1
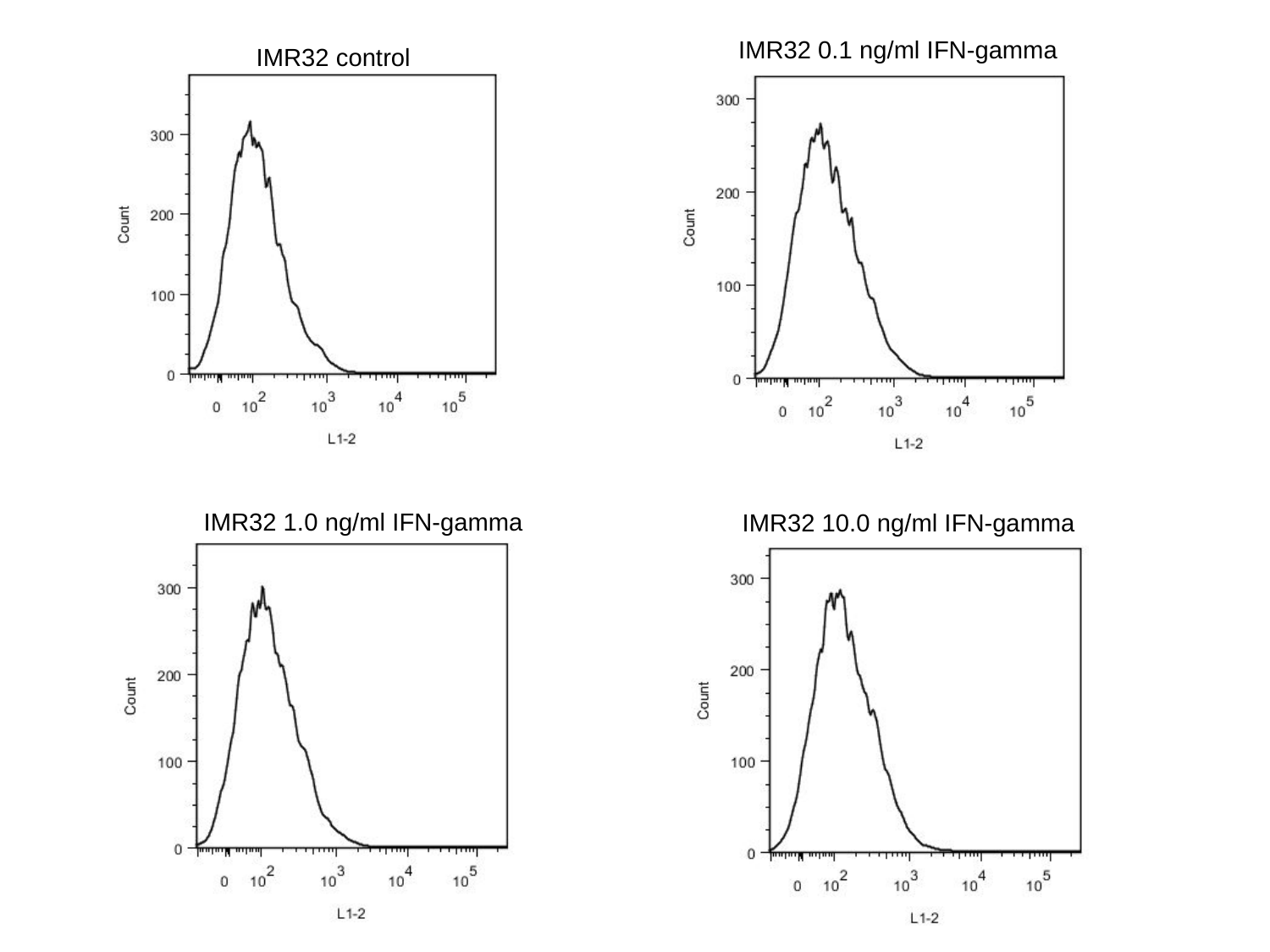

IMR32 0.1 ng/ml IFN-gamma
IMR32 control
IMR32 1.0 ng/ml IFN-gamma
IMR32 10.0 ng/ml IFN-gamma

Supplement: S5 Fig — (PPTX) [file pmed.1002497.s006.pptx]
