# Supplementary material for: PD-L1 checkpoint inhibition and anti-CTLA-4 whole tumor cell vaccination counter adaptive immune resistance: A mouse neuroblastoma model that mimics human disease
Source: PLoS Med. 2018 Jan 29;15(1):e1002497. doi: 10.1371/journal.pmed.1002497 (PMC5788338; doi:10.1371/journal.pmed.1002497)
Supplement: S3 Fig — N2a target cells are labeled with far-red dye. Killing of targets is detected by the shift in the combined far-red and caspase-positive population. (PPTX) [file pmed.1002497.s004.pptx]

## Slide 1
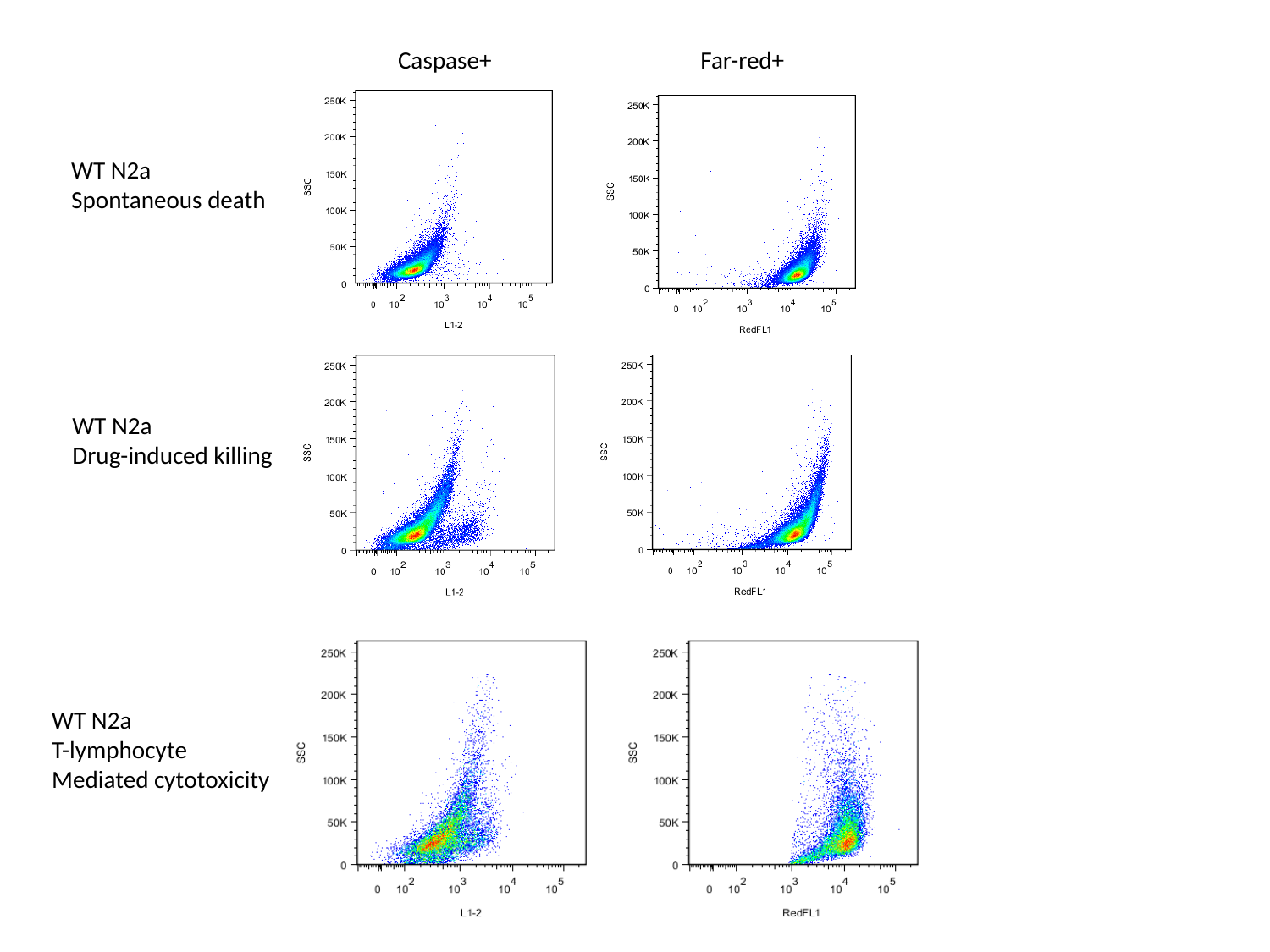

Caspase+
Far-red+
WT N2a
Spontaneous death
WT N2a
Drug-induced killing
WT N2a
T-lymphocyte
Mediated cytotoxicity

## Slide 2
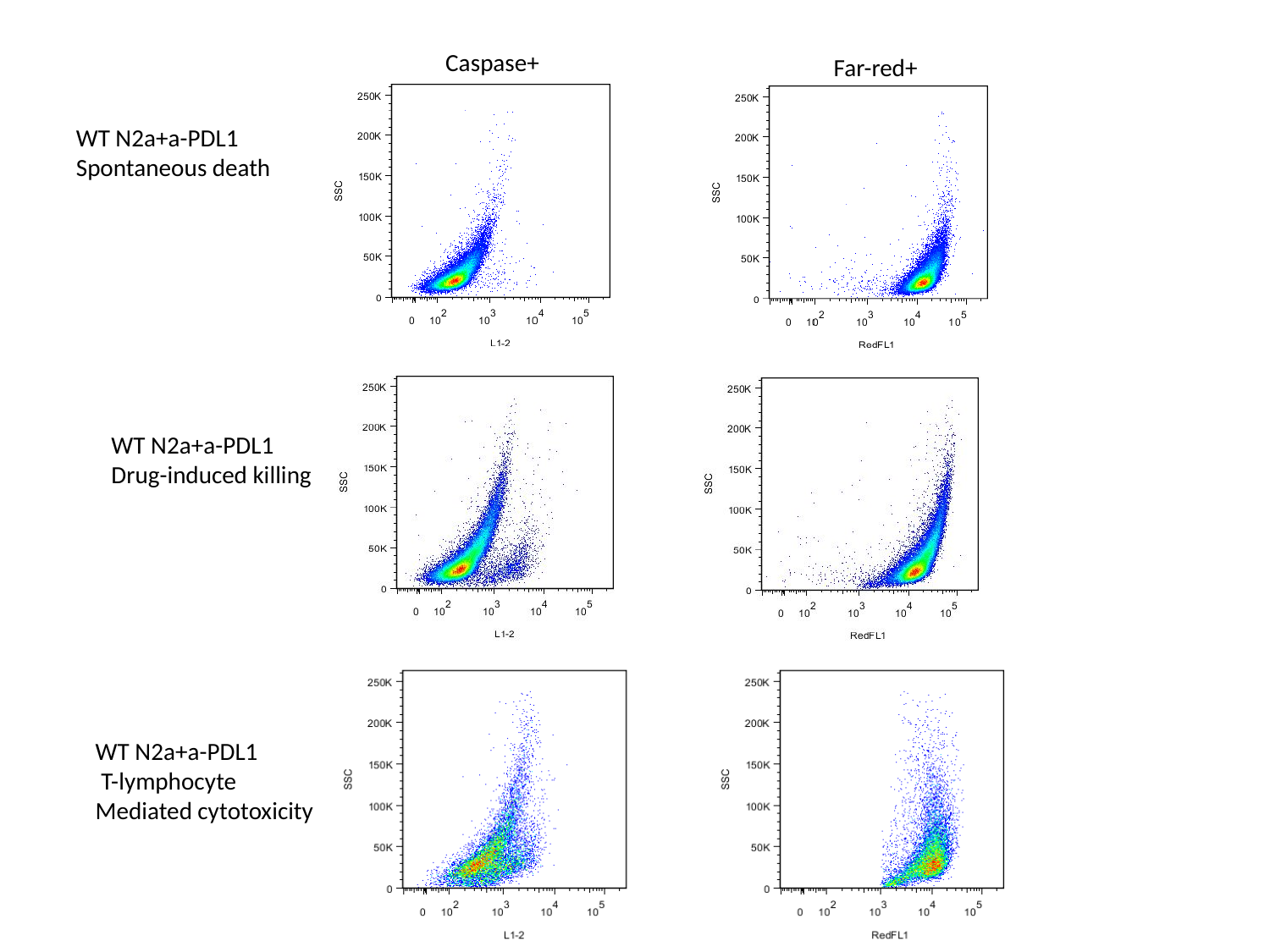

Caspase+
Far-red+
WT N2a+a-PDL1
Spontaneous death
WT N2a+a-PDL1
Drug-induced killing
WT N2a+a-PDL1
 T-lymphocyte
Mediated cytotoxicity

## Slide 3
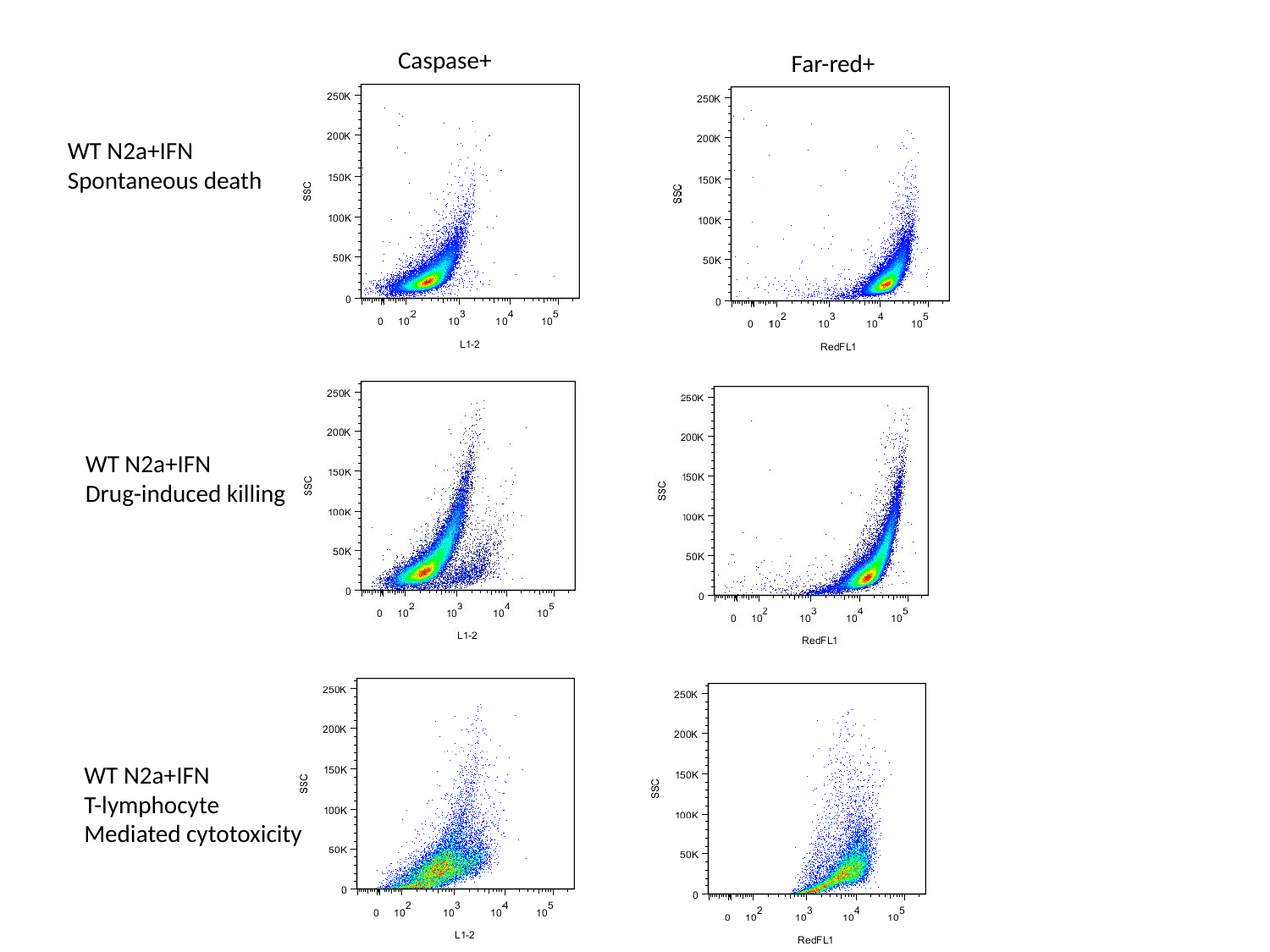

Caspase+
Far-red+
WT N2a+IFN
Spontaneous death
WT N2a+IFN
Drug-induced killing
WT N2a+IFN
T-lymphocyte
Mediated cytotoxicity

## Slide 4
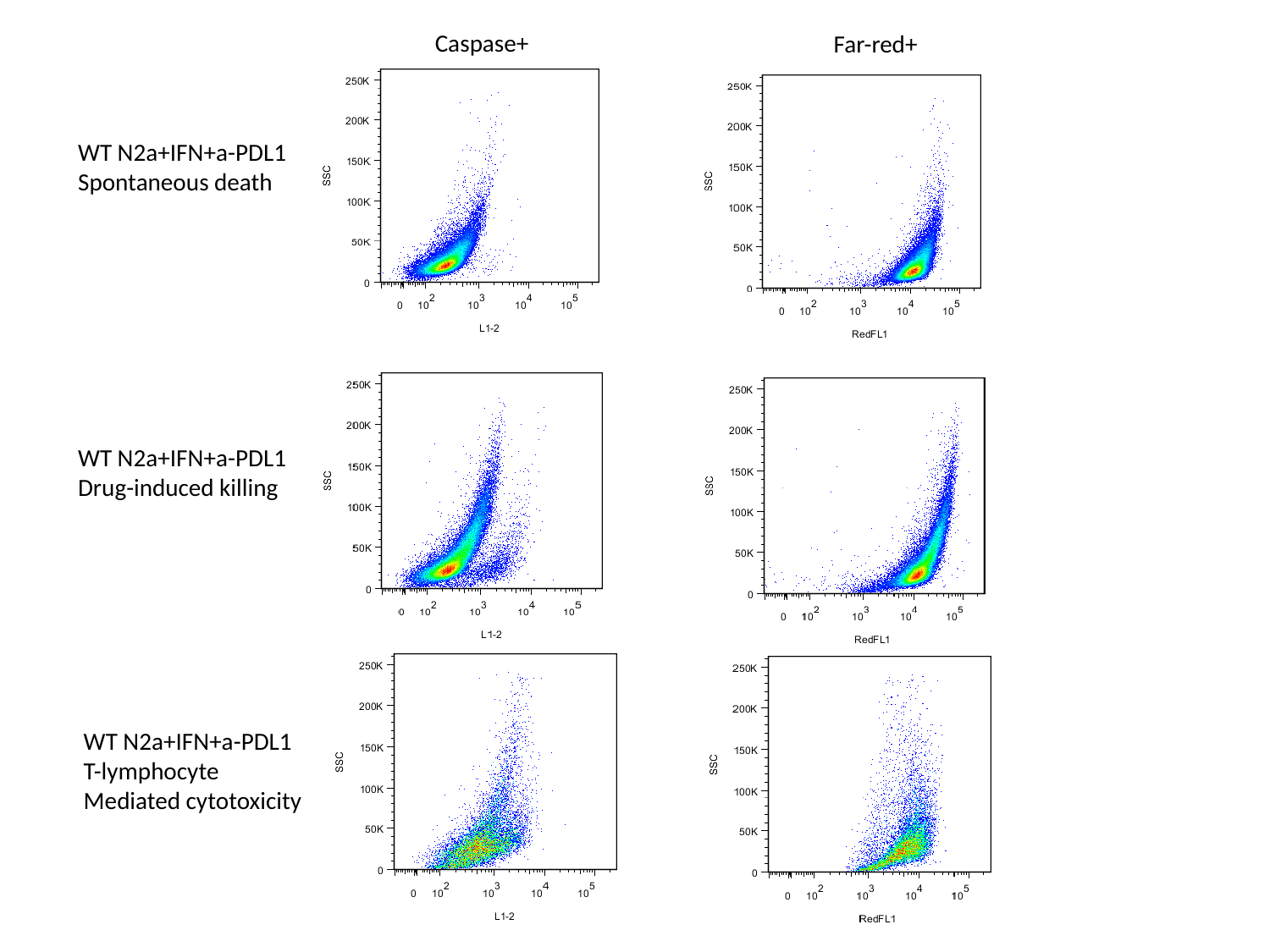

Caspase+
Far-red+
WT N2a+IFN+a-PDL1
Spontaneous death
WT N2a+IFN+a-PDL1
Drug-induced killing
WT N2a+IFN+a-PDL1
T-lymphocyte
Mediated cytotoxicity
